# Master mitotic kinases regulate viral genome delivery during papillomavirus cell entry

**DOI:** 10.1038/s41467-023-35874-w

**Published:** 2023-01-23

**Authors:** Matteo Rizzato, Fuxiang Mao, Florian Chardon, Kun-Yi Lai, Ruth Villalonga-Planells, Hannes C. A. Drexler, Marion E. Pesenti, Mert Fiskin, Nora Roos, Kelly M. King, Shuaizhi Li, Eduardo R. Gamez, Lilo Greune, Petra Dersch, Claudia Simon, Murielle Masson, Koenraad Van Doorslaer, Samuel K. Campos, Mario Schelhaas

**Affiliations:** 1 Institute of Cellular Virology, Westphalian Wilhelms-University of Münster, Münster, Germany; 2 Interfaculty Centre ‘Cells in Motion’ (CiM), Westphalian Wilhelms-University of Münster, Münster, Germany; 3 Max Planck Institute for Molecular Biomedicine, Münster, Germany; 4 Max Planck Institute of Molecular Physiology, Dortmund, Germany; 5 UMR 7242 Biotechnologie et signalisation cellulaire, CNRS, UdS, ESBS, Illkirch, France; 6 Institute of Medical Virology and Epidemiology of Viral Diseases, Tübingen, Germany; 7 School of Animal and Comparative Biomedical Sciences, University of Arizona, Tucson, AZ USA; 8 Department of Immunobiology, University of Arizona, Tucson, AZ USA; 9 Institute of Infectiology, Westphalian Wilhelms-University of Münster, Münster, Germany; 10 Cancer Biology Graduate Interdisciplinary Program, Genetics Graduate Interdisciplinary Program, UA Cancer Center, University of Arizona, Tucson, AZ USA; 11 Present Address: Department of Tropical Medicine, Medical Microbiology and Pharmacology, University of Hawai’i at Manoa, Honolulu, Hawaii 96813-5525 USA

**Keywords:** Human papilloma virus, Virus-host interactions, Mitosis

## Abstract

Mitosis induces cellular rearrangements like spindle formation, Golgi fragmentation, and nuclear envelope breakdown. Similar to certain retroviruses, nuclear delivery during entry of human papillomavirus (HPV) genomes is facilitated by mitosis, during which minor capsid protein L2 tethers viral DNA to mitotic chromosomes. However, the mechanism of viral genome delivery and tethering to condensed chromosomes is barely understood. It is unclear, which cellular proteins facilitate this process or how this process is regulated. This work identifies crucial phosphorylations on HPV minor capsid protein L2 occurring at mitosis onset. L2’s chromosome binding region (CBR) is sequentially phosphorylated by the master mitotic kinases CDK1 and PLK1. L2 phosphorylation, thus, regulates timely delivery of HPV vDNA to mitotic chromatin during mitosis. In summary, our work demonstrates a crucial role of mitotic kinases for nuclear delivery of viral DNA and provides important insights into the molecular mechanism of pathogen import into the nucleus during mitosis.

## Abstract (web summary)

Human papillomavirus (HPV) coopts mitosis for nuclear entry by tethering the viral DNA to mitotic chromosomes, a process facilitated by the viral minor capsid protein L2. Here, Rizzato et al. show that L2 contains conserved phosphorylation motifs within the chromosome-binding region and provide evidence that host master mitotic kinases CDK1 and PLK1 sequentially mediate phosphorylation of L2 at mitosis onset to allow timely tethering of viral DNA to mitotic chromosomes.

## Introduction

For initial infection, viruses must cross cellular barriers such as the plasma membrane or the nuclear envelope. This process is termed virus entry, where each step is tightly regulated by interactions of incoming viruses with cellular proteins^1^. The last step in the entry program is the delivery of viral genomes to the site of replication. While most viruses that replicate in the nucleus use nuclear pore complexes as delivery portals to the nuclear lumen, human papillomaviruses (HPVs) and certain retroviruses deliver their DNA after nuclear envelope breakdown during mitosis (NEBD)^2–6^. The molecular details of this unusual process are only poorly understood.

HPVs are a family of small non-enveloped DNA viruses of high clinical importance, since certain high-risk types cause anogenital and oropharyngeal malignancies including almost all cervical cancers^7–9^. Of the high-risk HPVs, HPV16 is the most prevalent and best-studied HPV type and serves as a paradigm for all HPVs.

The non-enveloped, icosahedral HPV capsids are formed by 72 pentameric capsomers of major capsid protein L1 and up to 72 molecules of minor capsid protein L2, and they enclose a circular, chromatinized double-stranded DNA genome (8 kb)^10–12^. HPVs initially infect mitotically active basal keratinocytes of the skin or mucosal epidermis, whereas its lifecycle is tightly linked to cell differentiation within the squamous epithelium^13–17^.

For HPV16, entry starts with binding to heparan sulfate proteoglycans^18^, which initiates structural changes within capsids that favor receptor switching and uptake by endocytosis^19–24^. The secondary receptor remains elusive but likely involves specifically functionalized tetraspanin-enriched microdomains, also termed HPV entry platforms^25,26^. Uptake itself occurs asynchronously over several hours by a novel actin-mediated endocytosis pathway^27^. Intracellular trafficking routes viruses to the endosomal pathway, where intralumenal low pH triggers uncoating and separates L1 in part from the viral DNA (vDNA)/L2 complex^19,28^. At this stage, L2 inserts into and spans the limiting membrane via its C-terminus^29–31^. It has been proposed that a large portion of L2 is exposed cytosolically and enables interactions with cellular trafficking factors such as the retromer complex, which directs viruses to the trans Golgi network^29,32–34^.

Later steps leading to intranuclear delivery remain poorly understood. Viruses remain within the Golgi until cell cycle progression into mitosis^6,35^, which likely helps to avoid intracellular immune sensing^36–38^. Upon mitosis onset, NEBD facilitates access of HPV to the nuclear lumen^5,39^. During prometaphase, the vDNA/L2 complex transits from the Golgi to microtubule-organizing centers likely still enclosed in a vesicle before reaching mitotic host chromatin^4,36,40^. Transit occurs along mitotic spindle microtubules through interaction with cellular adaptors and microtubular motors^41–43^. Viruses are tethered to mitotic chromatin, and remain associated until reformation of nuclear envelopes, which is phenocopied by L2 itself by associating with mitotic chromatin^44^. Tethering is mediated through interaction of the chromosomal-binding region (CBR) of L2 with unknown cellular factors^4,5^. Within the CBR, several conserved amino acid motifs are crucial for tethering, nuclear import, and infection^4^. Thus, a mode of nuclear delivery of HPV vDNA emerges. However, to date we do not understand the interactions and regulatory triggers that facilitate nuclear delivery mechanistically.

Regulation of mitotic events is achieved by reversible phosphorylations mediated by master mitotic kinases. These include cyclin-dependent kinases (CDKs), Polo-like kinases (PLKs), and Aurora kinases (AURKs), which act in concert by differentially phosphorylating their substrates in space and time^45,46^. The CDK1:CyclinB-CKS1 complex dictates entry into mitosis by promoting nuclear envelope breakdown, Golgi fragmentation, and assembly of the mitotic spindle, among others^47–50^. Concomitantly, PLK1 coordinates Golgi fragmentation, centrosome maturation, and kinetochore-microtubules attachment during mitosis^51–53^. Importantly, CDK1 activation requires the activity of PLK1, and the two serine/threonine kinases act in concert to coordinate mitotic events^54–57^.

Here, we identify another highly conserved motif (SSTP 212–215) of L2 and its role during nuclear import. As a well-known mitotic phosphorylation motif^45^, this SSTP sequence emerged as a regulatory site for HPV nuclear import through phosphorylation of L2 by mitotic kinases involved in coordinating proper chromosomal and organelle inheritance during mitosis. As such, phosphorylation of L2 by mitotic kinases provides a crucial trigger and activator for timely nuclear import of HPVs during mitosis. Hence, papillomaviruses evolved to coopt a cellular mechanism to synchronize their own nuclear entry. This example may serve as a paradigm for other viruses entering the nucleus via the same route.

## Results

### A highly conserved SSTP motif within L2 is required for the association to host cell chromatin

**Fig. 1:**
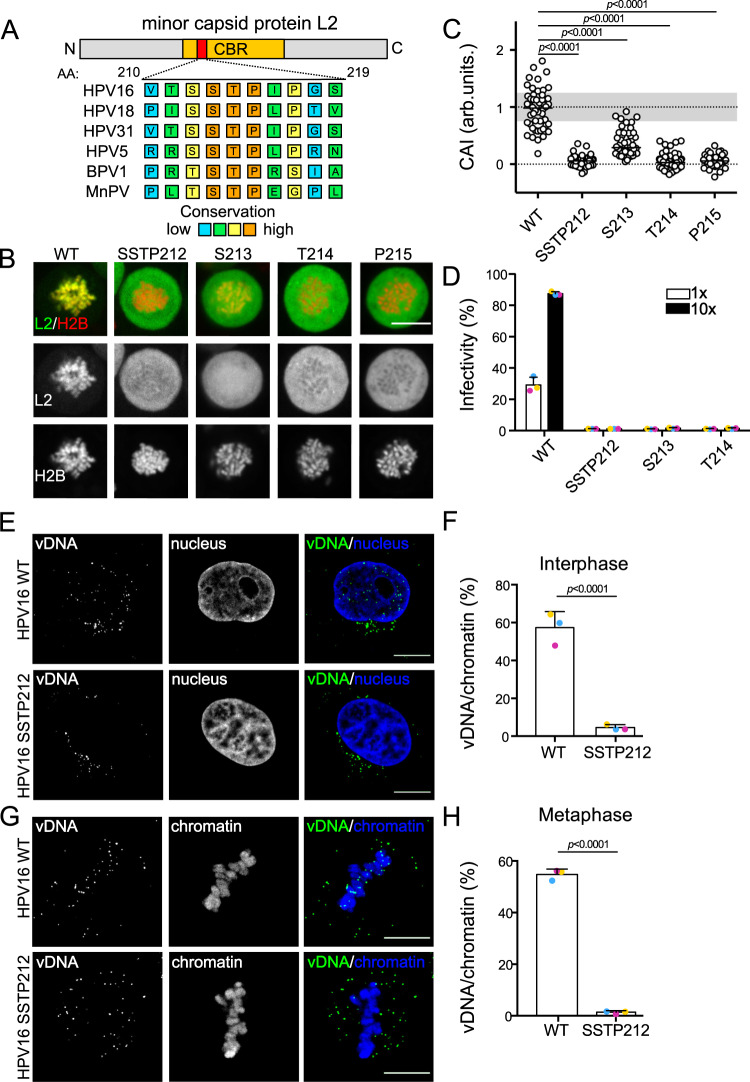
A highly conserved SSTP motif on HPV16-L2 is essential for the chromosomal association during mitosis. **A** Alignment of minor capsid protein L2 sequences for the indicated human and animal PVs (UniProtKB P03107, P06793, P17389, P06918, P03109, and Q84358) was generated using PRALINE (Bawono and Heringa, 2014). **B** Chromosomal association assay of ectopically expressed wild type and mutant L2-EGFPs in HeLa H2B-mCherry cells during mitosis; images display representative single medial planes of spinning disk confocal microscopy with L2-EGFP in green and H2B-mCherry in red as indicated. Scale bar: 10 µm. **C** Quantification of **B**, displaying the chromosomal association index (CAI) of individual cells (circles), i.e., the intensity of H2B-mCherry-overlapping EGFP signal over total intensity^4^. Values were normalized to EGFP alone (0) and L2-GFP (1). 50 cells from three independent experiments were analyzed. **D** HeLa cells were infected with L2-WT and mutant HPV16 PsVs as indicated. Infectivity was assessed 48 h post infection (p.i.) by scoring GFP-positive cells by flow cytometry. Virus amounts correspond to 1× = 25 ng L1, 10× = 250 ng L1. Displayed is the average of three independent experiments ± standard deviation (SD). **E**, **G** HeLa cells were infected with WT and SSTP212AAAA L2 mutant HPV16-EdU PsV for 20 h. Displayed are representative medial confocal slices of the subcellular localization of vDNA (EdU, green) and nuclei (Hoechst–blue) in interphase (**E**) and mitotic **G** cells. Scale bars: 5 µm. **F** Quantification of **E**, and **H** quantification of **G** for three independent experiments with eight cells/experiment. The overlap of vDNA/chromatin was quantified using intensity-based colocalization analysis (IMARIS Coloc function). Displayed is the average of three independent experiments ± SD. For all bar graphs, colored dots represent data points of individual experiments. For all quantifications, statistical significance was assessed by two-tailed Student’s *t* test to wildtype (WT). Source data are provided as a Source Data file.

The CBR of L2 is sufficient and required for chromosome tethering of incoming vDNA during mitosis^4^. In its N-terminal region, we identified an SSTP motif [AA 212–215], which is conserved among animals and HPVs (Fig. 1A). Since STP motifs are frequently targets of phosphorylation during mitosis^45^, we assessed its potential importance for nuclear delivery. First, an L2 mutant with four alanine substitutions of the SSTP motif (SSTP212AAAA) and tagged with EGFP were generated and subjected to a chromosomal association assay in prometaphase-arrested cells to test whether they would impair association. As expected, wildtype (WT) L2 overlapped with condensed cellular chromatin indicating its association (Fig. 1B)^4^. In contrast, L2-SSTP212AAAA did not associate (Fig. 1B). Quantitatively, the association is reflected by the chromosomal association index (CAI), i.e., the intensity of chromatin-associated over total cellular signal (Fig. 1C)^4^. While WT L2 showed maximal overlap (CAI = 1), L2 S213A exhibited drastically reduced overlap (CAI = 0.3), whereas L2-SSTP212AAAA, T214A, and P215A completely failed to associate (CAI = 0). Mutations in analogous sites of HPV18, HPV5, and bovine papillomavirus type 1 (BPV1) L2 replicated this result (Supplementary Fig. 1). This implied that an intact S/TSTP motif is required for L2 chromosomal association during mitosis for papillomaviruses of different genera.

### HPV16-L2 SSTP mutants are not infectious and fail in nuclear delivery

To ascertain that the SSTP motif was relevant for HPV16 entry, pseudoviruses (PsVs) harboring WT L2 or L2-SSTP212AAAA, S213A, and T214A were generated. While all HPV16-L2 mutants correctly assembled into virions (Supplementary Fig. 2), they failed to infect HeLa cells or HaCaT keratinocytes in stark contrast to WT HPV16 (Fig. 1D, Supplementary Fig. 3A) demonstrating that the SSTP motif of L2 is essential for HPV16 entry. Moreover, mutating the analogous S/TSTP sites of HPV18 and BPV1 also abrogated PsV infection of HeLa cells indicating this motif’s importance for additional papillomavirus types (Supplementary Fig. 3B, C).

Since L2 associates with and directs vDNA to mitotic chromosomes^4–6^, and since the L2 mutants did not associate with mitotic chromosomes (Fig. 1C), the inability to infect cells was likely caused by impaired tethering of vDNA to mitotic chromatin and, in consequence, failed nuclear delivery. HPV16 vDNA is detectable within nuclei typically 24 h post infection (p.i.) (Fig. 1E). At this time, about 60% of cell-associated vDNA localized to cell nuclei after infection with WT HPV16 (Fig. 1F). In case of HPV16-L2-SSTP212AAAA, however, only about 5% overlap was observed confirming that nuclear delivery of vDNA is impaired upon disrupting the SSTP motif (Fig. 1F). In mitotic cells, about 55% of vDNA localized to mitotic chromatin upon WT HPV16 infection indicating successful tethering (Fig. 1G, H). Notably, almost no vDNA was detectable on mitotic chromatin upon HPV16-L2-SSTP212AAAA infection (Fig. 1G, H) implying that the SSTP motif is crucial for tethering vDNA to mitotic chromatin. To rule out any entry defects of these mutants prior to nuclear import, delivery of vDNA to the Golgi apparatus was assessed by colocalization analysis. For both, WT and SSTP mutant HPV16, vDNA accumulated in the Golgi to similar extents (Supplementary Fig. 3D, E) confirming that the impairment in entry occurred post-Golgi delivery.

Previous work using virion-incorporated C-terminal L2-BirA biotin ligase fusion proteins indicated that the L2 C-terminus becomes accessible for cytosolic GFP-BAP (biotin acceptor peptide) substrate biotinylation upon mitosis^40^, and that mutations within the CBR, which impair nuclear delivery, also prevent proximity biotinylation^4,40^. Using this assay as an alternative assay for post-Golgi steps and nuclear delivery, incoming HPV16 harboring L2-BirA SSTP212AAAA failed to biotinylate GFP-BAP in contrast to WT HPV16 supporting a pivotal role for this motif in post-Golgi steps (Supplementary Fig. 4A, B).

### HPV16-L2 is phosphorylated on the SSTP [212–215] motif during mitosis

**Fig. 2:**
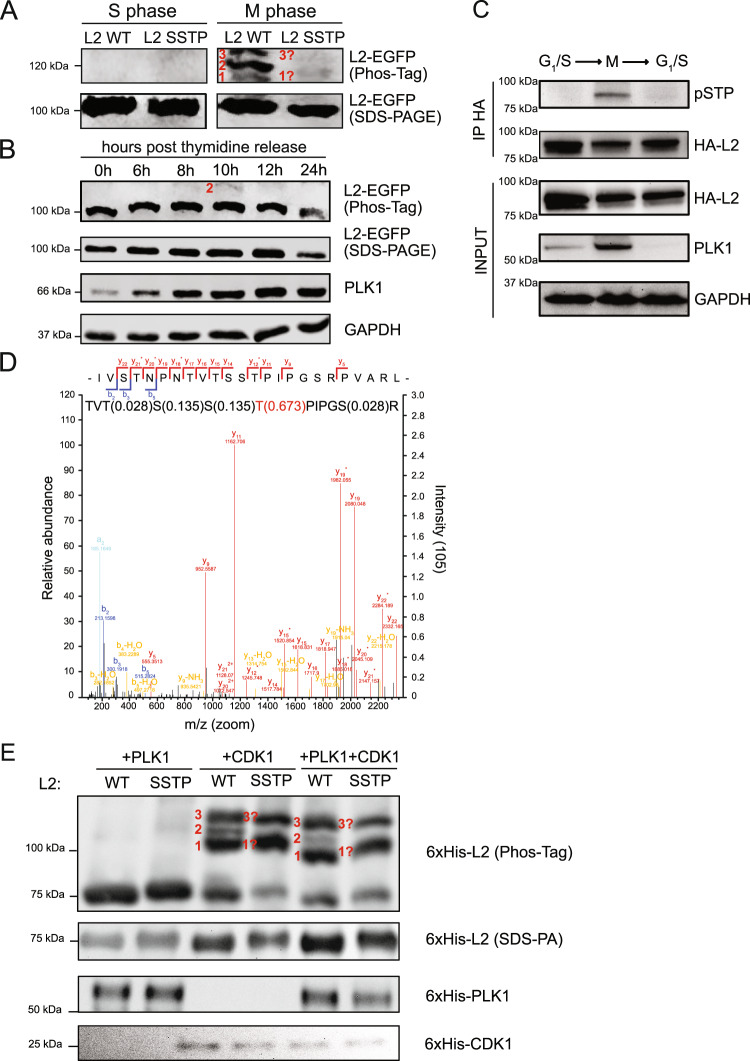
L2 is phosphorylated by CDK1 and PLK1 during mitosis. **A** HeLa cells were transfected with WT and SSTP212AAAA L2-EGFP expression constructs, and subsequently arrested in G_1_/S- or M-phase using aphidicolin (3 µM) or nocodazole (330 nM), respectively. Depicted is a representative example of five independent experiments from Western blot analysis against L2 (Santa Cruz #sc-65709) of SDS-PA and Phos-Tag gel electrophoresis of cell lysates. Red numbers annotate different L2 phosphorylation bands. **B** As in **A**, but with L2-EGFP only. HeLa cells were arrested in S-phase by double thymidine block. Cell lysates from indicated time points after block release were subjected to analysis as in **A**. Depicted is a representative example of five independent experiments from Western blot analysis of SDS-PA and Phos-Tag gel electrophoresis. Red numbers annotate different L2 phosphorylation bands. **C** HEK293 cells were transfected with a WT L2-3xHA expression construct. Subsequently cells were arrested in G_1_/S-phase (left), in mitosis after double thymidine arrest in S-phase and release into nocodazole (M, middle), or in the subsequent G_1_/S-phase (right). L2 was immunoprecipitated from cell lysates (input) using an HA antibody and analyzed by Western blotting against indicated targets. Depicted is a representative example of three independent experiments. **D** HEK293 cells were transfected with WT and SSTP212AAAA L2-3xHA constructs, arrested in mitosis or G_1_/S by nocodazole (330 nM) and aphidicolin (3 µM), and subjected to immunoprecipitation (IP) using an anti-HA.11 antibody (BioLegend #901516). IPs were subsequently prepared for mass spectrometry analysis. Depicted is a representative LC-MS/MS spectrum of mitotic L2-3xHA peptides of three independent experiments containing the phosphorylated L2 SSTP motif; numbers between parenthesis represent phosphorylation probabilities of residues. **E** In vitro kinase assay of WT and SSTP212AAAA purified 6xHis-L2 in presence of 6xHis-PLK1, or of 6xHis-CDK1-CyclinB:CKS1 (CCC) complex, or of both kinases. Depicted are representative examples from Western blot analysis against L2 (Santa Cruz sc-65709), PLK1 (Abcam ab17057) and CDK1 (Santa Cruz #sc-54) of SDS-PA and Phos-Tag gel electrophoresis of five independent in vitro kinase assays. Red numbers indicate differential phosphorylation states of L2. Source data are provided as a Source Data file.

Next, we asked how the SSTP motif may contribute to nuclear delivery. A STP motif constitutes a consensus sequence for phosphorylation by mitotic kinases such as cyclin-dependent and mitogen-activated protein kinases^58–60^. In addition, T/S-S-p(T)-P is a binding consensus motif of PLKs bearing functional Polo-box-domains (PBDs)^61,62^. Since L2 can be post-translationally modified by phosphorylations^63–65^, the SSTP motif may be a phosphorylation site. To address whether L2 would be phosphorylated during mitosis, L2 was subjected to Phos-Tag gel electrophoresis after ectopic expression in G_1_/S- or prometaphase-arrested cells (Fig. 2A). Phos-Tag gel electrophoresis leads to prominent electrophoretic mobility shifts of phosphorylated proteins. In mitotic cell lysates, three additional bands for WT L2 appeared in comparison to lysates from G_1_/S-arrested cells (Fig. 2A, bands 1, 2, 3). Notably, L2-SSTP212AAAA displayed no such shifts or only weak remnants of two of these bands (Fig. 2A, bands 1, 3) indicating that the SSTP site is phosphorylated during mitosis. Phosphorylation of the SSTP site was supported by pSTP signals that occurred in mitotic but not G_1_/S-arrested cells and only for WT but not mutant L2 after immunoprecipitation (Supplementary Fig. 5A). To shed light on L2 phosphorylation during the cell cycle, the dynamics of L2 phosphorylation were characterized after cell cycle synchronization by thymidine arrest in and release from the synthesis (S) phase. Chasing L2 phosphorylation after thymidine release, Phos-Tag analysis showed that it started at 8 h, remained until 12 h, and disappeared at 24 h post release (Fig. 2B, band 2). Here, only one band was easily detectable. This suggested phosphorylation of only the main site was observed, likely because only a fraction of cells were in mitosis at certain times after release in contrast to the majority of cells upon mitotic arrest (compare Fig. 2A). Thus, L2 phosphorylation was a dynamic process, which occurred during mitosis, and decreased after cytokinesis indicating a cell cycle-dependent phosphorylation of HPV16-L2 during G2/M phases, when the vDNA is delivered onto mitotic chromatin by L2^4^.

To confirm that the L2 SSTP motif was phosphorylated during mitosis and dephosphorylated after mitosis was completed, we probed pSTP of immunoprecipitated L2 during cell cycle arrest. STP phosphorylation was not detectable in G_1_/S-arrested cells, occurred in mitotic cells, but vanished again, if cells were released from a mitotic block into a G1/S arrest (Fig. 2C), SSTP212AAAA mutation prevented pSTP signals during mitosis arrest (Supplementary Fig. 5A), confirming dynamic phosphorylation and dephosphorylation of the L2 SSTP site during the cell cycle. In addition, label-free mass spectrometry was employed. Indeed, phosphorylation of the SSTP motif, mainly on threonine 214, was detected exclusively in samples from mitotic but not G_1_/S-arrested cells (Fig. 2D) as expected by the absence of phosphorylation in L2 in G_1_/S-arrested cells (Figs. 2A, C).

### CDK1 primes the SSTP motif for PLK1 binding

As L2 was phosphorylated on its SSTP site, we hypothesized that it could be a docking site for mitotic PLK1. PLK1 consists of a N-terminal globular kinase domain and C-terminal PBD (reviewed in ref. ^62^). The polo-boxes form an intramolecular dimer that specifically binds phospho-substrates with the consensus sequence S-p(S)/p(T)-P (pSTP), the canonical CDK1 phosphorylation sequence, thereby regulating PLK1 subcellular localization and kinase activity^66^.

To assess the role of CDK1 and PLK1 in phosphorylation more directly, we used an in vitro kinase assay. For this, recombinant purified and refolded HPV16-L2 was incubated with recombinant active CDK1:CyclinB-CKS1 and/or PLK1 in presence of Mg^2+^-ATP. Phos-Tag analysis revealed electrophoretical shifts of L2 for both WT L2 and L2-SSTP212AAAA indicating that phosphorylation occurred (Fig. 2E). While PLK1 alone only weakly, if at all, phosphorylated L2, CDK1 strongly phosphorylated both WT L2 and L2-SSTP212AAAA. Notably, three major phospho-bands appeared for WT L2, whereas only two phospho-bands were apparent for L2-SSTP212AAAA. This finding suggested that the missing phosphorylation occurred at the SSTP motif (Fig. 2E, compare bands 1, 2, 3 with 1, 3). If L2 was incubated with both kinases, no obvious additional bands appeared (Fig. 2E). Thus, the SSTP motif was likely phosphorylated by CDK1.

Since Phos-Tag analysis exclusively indicates the presence of major phosphorylations, we verified our conclusions. pSTP of in vitro phosphorylated L2 was observed exclusively in presence of CDK1 for WT but not SSTP212AAAA L2 (Supplementary Fig. 5B) strongly supporting phosphorylation of the L2 SSTP motif by CDK1. This also implied that additional phospho-bands of L2-SSTP212AAAA upon CDK1 exposure (compare Fig. 2D) were not bona fide pSTP sites. We further adopted mass spectrometry analysis of in vitro phosphorylated WT or SSTP mutant L2 to identify all potential phosphorylation sites. It is important to note, however, that in vitro phosphorylations may occur to an extent that exceeds the degree or sites of phosphorylations occurring in vivo. Our analysis revealed several different phosphorylations of L2 in presence of CDK1 or PLK1 alone. Importantly, phosphorylation at the SSTP site was detectable only in presence of CDK1 confirming that CDK1 indeed can mediate phosphorylation at this site (Supplementary Table 1). Intriguingly, three phosphorylation sites were identified only if WT but not mutant L2 was incubated in presence of both kinases, namely T209, T265, and S319 (Supplementary Table 1). This would mean that PLK1 was able to phosphorylate L2 at these sites only if CDK1 primed PLK1 binding to phosphorylated SSTP sites. Of note, phosphorylated T265 was also observed upon immunoprecipitation of ectopically expressed L2 in mitotic cell lysates (assay of Fig. 2D). Taken together, these results provide evidence that L2 was phosphorylated by both CDK1 and PLK1 likely in a sequential fashion, reminiscent of cellular CDK1 and PLK1 targets during mitosis.

### PLK1 interacts with HPV16-L2 via the SSTP motif

**Fig. 3:**
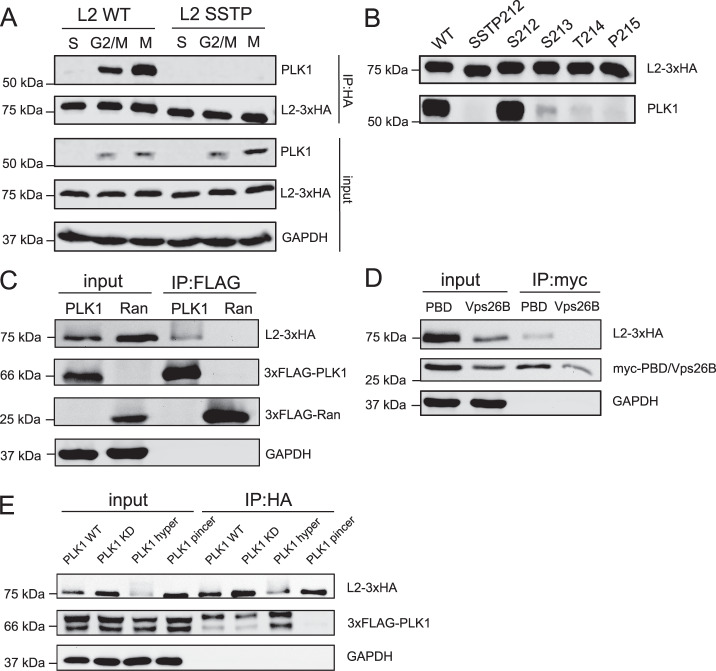
L2 SSTP motif is essential for interaction with PLK1 through the Polo-box domain. **A** HEK293 cells were transfected with WT and SSTP212AAAA mutant L2-3xHA expression constructs, and subsequently arrested in S-phase, M-Phase or at the G2/M border using aphidicolin (3 µM), nocodazole (330 nM) or RO-3306 (9 µM). Depicted is a representative example from Western blot analysis against HA tag (BioLegend #901516), PLK1 (Abcam ab17057), or GAPDH (Proteintech #10494-1-AP) as loading control of SDS-PA gel electrophoresis of endogenous PLK1 immunoprecipitates (Abcam ab17057). **B** HEK293 cells were transfected with WT and SSTP212AAAA, S213A, T214A or P215A L2-3xHA expression constructs, and subsequently arrested in M-Phase using nocodazole (330 nM). Depicted is a representative example from Western blot analysis against HA tag (BioLegend #901516), and endogenous PLK1 (Abcam ab17057), of SDS-PA gel electrophoresis of PLK1 immunoprecipitates (Abcam ab17057). **C** HEK293 cells were co-transfected with WT L2-3xHA and 3xFLAG-PLK1 or 3xFLAG-Ran expression constructs. Depicted is a representative example from Western blot analysis against L2 (Santa Cruz sc-65709), against FLAG tag for 3xFLAG-PLK1 and 3xFLAG-Ran (Sigma-Aldrich #F1804), against GAPDH (Proteintech #10494-1-AP) and of SDS-PA gel electrophoresis of FLAG tag PLK1 and Ran immunoprecipitates (Sigma-Aldrich #F1804). **D** HEK293 cells were co-transfected with WT L2-3xHA and myc-PBD or myc-Vps26B constructs. Depicted is a representative example from Western blot analysis against L2 (Santa Cruz sc-65709), against myc tag for PBD and Vps26B (ThermoFisher #13-2500), against GAPDH (Proteintech #10494-1-AP) and of SDS-PA gel electrophoresis of myc tag PBD and Vps26B immunoprecipitates (ThermoFisher #13-2500). **E** HEK293 cells were co-transfected with 3xHA-L2 and 3xFLAG-PLK1 constructs (WT: wild type, KD: kinase dead K82R, hyper: hyper activated T210D, pincer: Polo-box domains dead, H538A K540M). Depicted is a representative example from Western blot analysis against L2 (Santa Cruz sc-65709), against FLAG tag for 3xFLAG-PLK1 (Sigma-Aldrich #F1804), against GAPDH (Proteintech #10494-1-AP) and of SDS-PA gel electrophoresis of L2-3xHA (Santa Cruz sc-65709) and 3xFLAG-PLK1 (Sigma-Aldrich #F1804), immunoprecipitates. Representative blots for three independent experiments are shown. Source data are provided as a Source Data file.

We next assessed whether L2 and PLK1 would interact through the SSTP site as inferred by our previous experiments. Indeed, endogenous PLK1 co-immunoprecipitated with L2 during mitosis, but not during G_1_/S-phase, whereas L2-SSTP212AAAA failed to interact with PLK1 (Fig. 3A). Moreover, while L2 S213A partially retained interaction with PLK1, L2 mutations T214A and P215A abolished it similar to L2-SSTP212AAAA (Fig. 3B). In reverse, 3xFLAG-PLK1 immunoprecipitated L2 but not 3xFLAG-Ran that was used as negative control (Fig. 3C) further confirming the specificity of interaction. Of note, L2 failed to immunoprecipitate CDK1 (Supplementary Fig. 6) in line with a more commonly observed kiss-and-run interaction of kinases with their substrates suggesting that CDK1-L2 interactions are more transient than PLK1-L2 interactions. These findings, together with published data on PLK1 substrate binding^67^, strongly suggested that the L2 SSTP motif acted as a PLK1-binding phosphosite. If so, the PDB of PLK1 most likely mediated the interaction. Indeed, PBD-myc immunoprecipitated L2-3xHA (Fig. 3D) implying that PLK1 interacts with L2 through its PBD. To verify this notion and to test, whether PLK1’s kinase activity would be required for interaction, we generated PLK1 kinase dead (KD – K82R), PLK1 kinase hyperactive (hyper – T210D), and Polo-box dead (known as “pincer” - H538K M540R) mutants^68,69^. As expected, L2 interacted with all PLK1 versions but the Polo-box dead PLK1 (Fig. 3E) confirming that the interaction was mediated by the PBD. In contrast, the L2 SSTP mutant could not significantly interact with PLK1 (Supplementary Fig. 7A). Using Gaussia luciferase complementation as an orthogonal method to study protein-protein interactions^70^, the results were not only confirmed, but importantly demonstrated that L2 from different HPV types interacted with PLK1, indicating a conserved feature amongst the papillomaviruses (Supplementary Fig. 7B, C). Hence, PLK1 interacted with L2’s SSTP site via the PBD reminiscent of cellular substrates.

### PLK1 kinase activity is required for L2 tethering to the host chromatin during mitosis

**Fig. 4:**
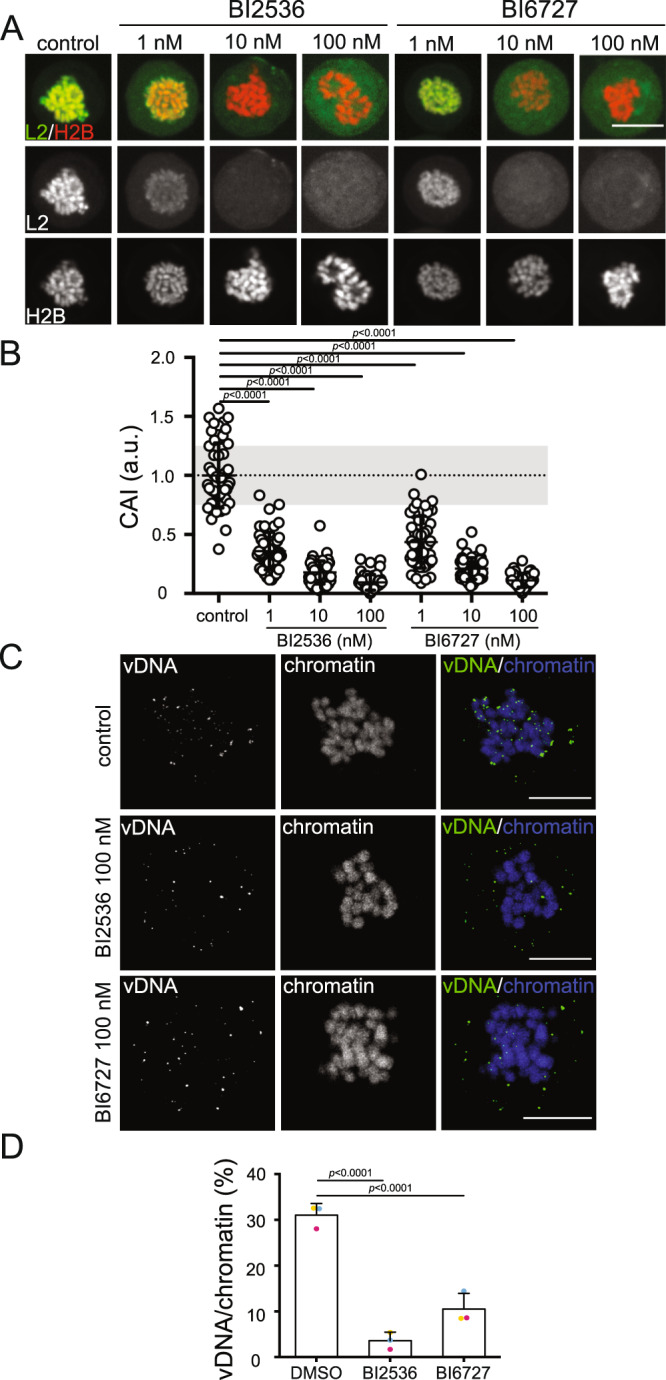
PLK1 inhibition impairs HPV16-L2 chromosomal association and vDNA delivery to host mitotic chromatin. **A** HeLa cells stably expressing L2-EGFP /H2B-mCherry were incubated in presence of the indicated PLK1 inhibitors (100 nM) for 16 h, arrested in mitosis using nocodazole (330 nM), and processed for chromosomal association assay as in Fig. 1B. Depicted are representative single medial planes of spinning disk confocal microscopy with L2-EGFP in green and H2B-mCherry in red as indicated. Scale Bar: 10 µm. **B** Quantification of **A**, displaying the chromosomal association index (CAI) of individual cells (circles), i.e. the intensity of H2B-mCherry-overlapping EGFP signal over total intensity^4^. Values were normalized to EGFP alone (0) and L2-GFP (1). 50 cells from three independent experiments were analyzed. Displayed is the average of three independent experiments ± SD. **C** HeLa cells were infected with WT L2 HPV16-EdU PsV for 20 h, then released into mitosis in presence of nocodazole (330 nM – control), nocodazole and BI2536 (100 nM), or nocodazole and BI6727 (100 nM). Displayed are representative medial confocal slices of the subcellular localization of vDNA (EdU, green) and nuclei (Hoechst - blue) in mitotic cells. Scale bars: 5 µm. **D** Quantification of **C** for three independent experiments with eight cells/experiment. The overlap of vDNA/chromatin was quantified using intensity-based colocalization analysis (IMARIS Coloc function). Displayed is the average of three independent experiments ± SD. Colored dots represent data points of individual experiments. Statistical significance was assessed by two-tailed Student’s *t* test to control. Source data are provided as a Source Data file.

Our data thus far implies that CDK1 phosphorylation of the L2 SSTP motif during mitosis provides a docking site for PLK1 to further phosphorylate L2, which, in turn, results in targeted delivery of L2 and vDNA to mitotic chromatin. To probe this hypothesis further, the role of PLK1’s activity in L2 chromosomal association was first investigated using two kinase PLK1 inhibitors, BI2536, and BI6727. Both, BI2536, a pan-Polo-like kinases inhibitor, and BI6727, an inhibitor more specific for PLK1 itself, dose-dependently reduced L2 association with mitotic chromatin in nanomolar concentrations (Figs. 4A, B). As a control for inhibitory efficacy, phosphorylation of Ser46 of translationally controlled tumor protein (TCTP), an established PLK1 substrate, was quantified (Supplementary Fig. 8A, B). Similar to its effects on L2 chromosomal association, phosphorylation of TCTP dose-dependently decreased in presence of BI2536 (Supplementary Fig. 8B). Aurora A kinase activates PLK1^71^. Similar to the PLK1 kinase inhibitors, Aurora A kinase inhibition by MLN8237 reduced the chromosomal association of L2 at concentrations that hampered TCTP phosphorylation (Supplementary Fig. 8E–H). Furthermore, poloxin, an inhibitor of PBD-mediated PLK1 substrate binding^72^, interfered with L2 chromosomal association and TCTP phosphorylation (Supplementary Fig. 8C, D, G, H). In summary, these data demonstrated that both, substrate binding and kinase activity of PLK1, were required for tethering of L2 to mitotic chromatin.

For verification, the L2-BirA proximity biotinylation assay was used once again. And indeed, PLK1 inhibition by BI2536 and BI6727 impaired GFP-BAP biotinylation, suggesting PLK1 kinase activity to be involved in post-Golgi steps of the entry program (Supplementary Fig. 4C).

### Inhibition of PLK1 prevents HPV16 vDNA delivery to mitotic chromatin

Next, we aimed to confirm that PLK1 phosphorylation is indeed required for HPV16 vDNA delivery to mitotic chromatin. For this, cells arrested at the G_2_-/M-phase with RO-3306, a reversible CDK1 inhibitor^73^, were infected with HPV16 harboring EdU-labeled vDNA. Subsequently, cells were released from G_2_/M into nocodazole-mediated prometaphase arrest in presence or absence of PLK1 inhibitors. In the control, ~30% of vDNA localized to mitotic chromatin (Figs. 4C, D). In contrast, vDNA localization to mitotic chromatin decreased significantly to ~4% and 12% upon PLK1 inhibition by BI2536 and BI6727, respectively, (Figs. 4C, D). These data are in line with a pivotal role of PLK1 kinase activity for HPV16 vDNA delivery to mitotic chromatin.

Of note, in G_1_/S-arrested cells, vDNA remained Golgi-associated after infection at least until 72h p.i., suggesting that it served as efficient ‘waiting room’ for HPV until cell cycle progression into mitosis and, thus, CDK1- and PLK1-mediated phosphorylations of L2 would occur (Supplementary Fig. 9).

### PLK1 phosphorylates L2 on the conserved T265 residue

**Fig. 5:**
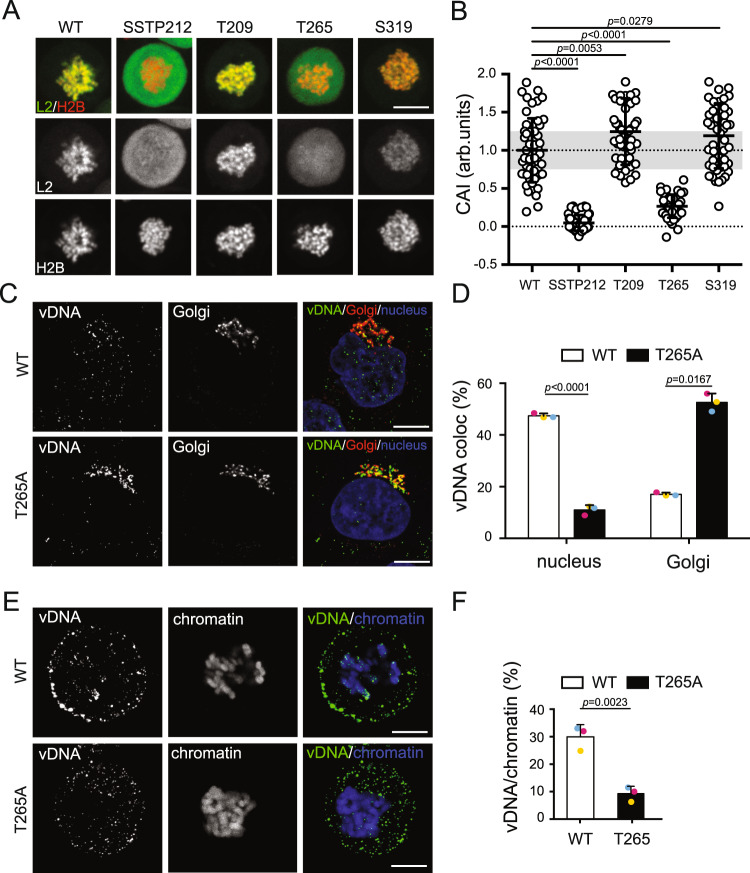
L2 T265 is a putative PLK1 phosphosite required for proper chromosomal association and vDNA tethering to host chromatin. **A** Chromosomal association assay of ectopically expressed wild type and T209A, T265A and S319A L2-EGFP in HeLa H2B-mCherry cells during mitosis. Images display representative single medial planes of spinning disk confocal microscopy with L2-EGFP in green and H2B-mCherry in red as indicated. Scale bar: 10 µm. **B** Quantification of **A**, displaying the chromosomal association index (CAI) of individual cells (circles), i.e., the intensity of H2B-mCherry-overlapping EGFP signal over total intensity^4^. 50 cells from three independent experiments were analyzed. Displayed is the average of three independent experiments ± SD. **C**, **E** HeLa cells were infected with WT and T265A L2 mutant HPV16-EdU PsV for 24 h. Images displayed are representative medial confocal slices of the subcellular localization of vDNA (EdU, green), Golgi (Giantin – red), and nuclei (Hoechst - blue) in interphase **C** and mitotic **E** cells. Scale bars: 5 µm. **D** Quantification of **C**, and **F** quantification of **E** for three independent experiments with eight cells/experiment. The overlap of vDNA/chromatin and vDNA/Golgi were quantified using intensity-based colocalization analysis (IMARIS Coloc function). Displayed is the average of three independent experiments ± SD. Colored dots represent data points of individual experiments. Statistical significance was assessed by two-tailed Student’s *t* test to wildtype (WT). Source data are provided as a Source Data file.

Three PLK1-dependent phosphosites within the L2 CBR were identified in the in vitro kinase assay, namely T209, T265, and S319 (Supplementary Table 1). This prompted us to assess which of these would be potential targets of PLK1 in vivo. Of those, only T265 was predicted in silico to be a potential PLK1 target. Moreover, T265 is conserved among the PVs as a phosphorylatable residue (serine or threonine), and most importantly was also found in L2 after mass spectrometry of ectopically expressed L2 in mitotic cells. All three residues were mutated into alanine in L2-EGFP, and mutants were scored for chromosomal association in prometaphase-arrested cells (Figs. 5A, B). While L2 T209A and S319A associated with mitotic chromatin to a similar degree or perhaps slightly better than WT L2 (Fig. 5B), L2-T265A association was significantly decreased (CAI = 0.3) yet less prominently than the SSTP mutant, where association was abolished (CAI = 0). This indicated that T265 was an important PLK1 phosphosite within the L2 CBR. Of note, L2-T265A was phosphorylated by CDK1 in vitro similar to L2-WT indicating that T265A did not impair SSTP 212 phosphorylation in line with being a PLK1 phosphosite (Supplementary Fig. 5B).

To verify T265’s importance for HPV16 entry, nuclear delivery and chromatin association of vDNA during mitosis of HPV16 WT and T265A mutant were analyzed as before. For HPV16 WT, vDNA localized predominantly to the nucleus, whereas HPV16 T265A mutant mostly remained localized to the Golgi (Figs. 5C, D) indicating impaired nuclear but successful Golgi delivery. During mitosis, ~30% of vDNA localized to mitotic chromatin upon WT infection, whereas vDNA was associated only to ~10% for T265 mutant virions (Figs. 5E, F). Overall, these results indicated that the T265 phosphosite was important during entry implying that PLK1-mediated phosphorylation is another important regulatory step for efficient L2/vDNA delivery to mitotic chromatin.

### CDK1 phosphorylation is primarily required to recruit PLK1 to L2

**Fig. 6:**
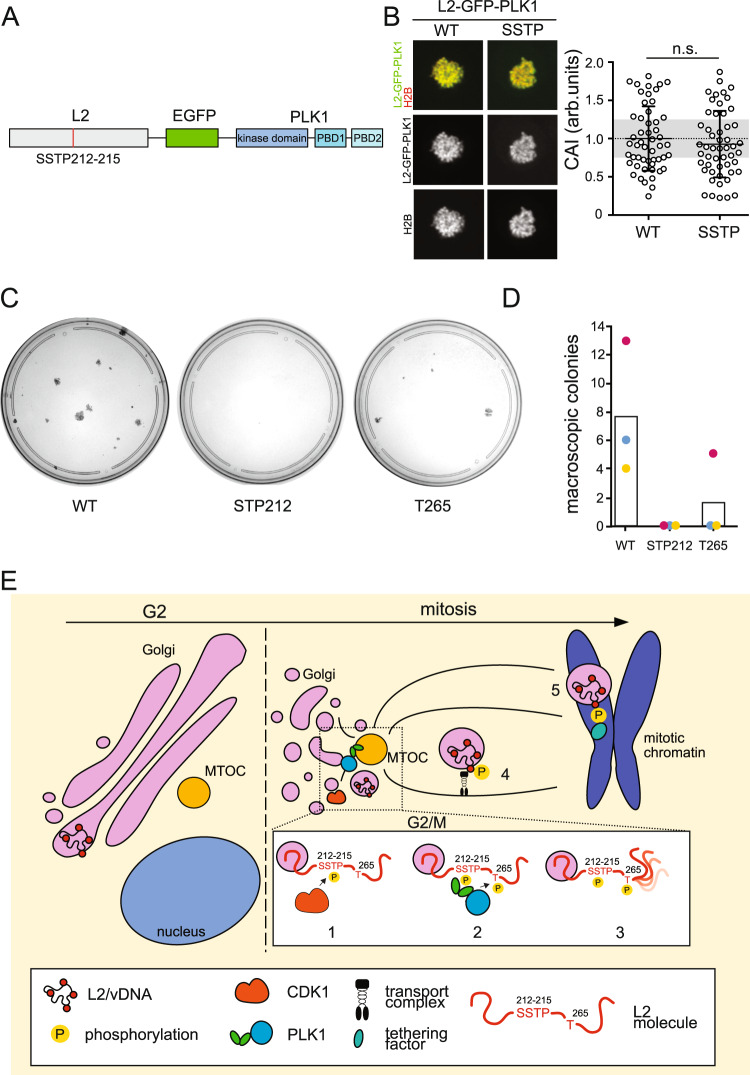
CDK1 phosphorylation is primarily required to recruit PLK1 to L2. **A** Schematic depiction of L2-GFP-PLK1 WT and SSTP212AAAA mutant chimeras generated to study chromosomal association. **B** Chromosomal association assay of ectopically expressed wild type and SSTP 212 L2-EGFP-PLK1 chimera in HeLa H2B-mCherry cells arrested with nocodazole (330 nM) in mitosis; images display representative single medial planes of spinning disk confocal microscopy with L2-EGFP-PLK1 in green and H2B-mCherry in red as indicated. The chromosomal association index (CAI) of individual cells (circles), i.e. the intensity of H2B-mCherry-overlapping EGFP signal over total intensity^4^. 50 cells from three independent experiments were analyzed. Displayed is the average of three independent experiments ± SD. Statistical significance was assessed by two-tailed Student’s *t* test. **C** Quantitative colony formation assay was used to test the ability of HPV18 genomes to persist in keratinocytes. Infected cells were treated with G418 for the duration of the experiment. Colonies were visualized using MMT. Representative images are shown. **D** Quantification of data shown in **C**. Three separate donors were used for the primary human keratinocytes. **E** Proposed model for sequential phosphorylation of L2 by master mitotic kinases CDK1 and PLK1, which regulate L2 delivery to mitotic chromosomes: L2 spanning across the Golgi limiting membrane allows for CDK1 phosphorylation at the SSTP motif (1), thus priming the viral protein for interaction with PLK1 PBD (2), resulting in further L2 phosphorylations, including the T265 residue (3). Phosphorylated L2, spanning from a bona fide Golgi-derived vesicle travels via MTs (4) and is tethered to mitotic chromosomes (5), promoted by the phosphorylation state, which allows interaction. Source data are provided as a Source Data file.

The kinase activities of CDK1 and PLK1 were essential for the timely and successful delivery of the L2/vDNA subcomplex to host mitotic chromatin. Since PLK1 appeared to bind strongly and persistently to L2, we wondered whether CDK1’s role would be primarily to recruit PLK1 by phosphorylating the SSTP site. Thus, we generated L2-GFP-PLK1 fusion proteins with or without the SSTP mutation (Fig. 6A) to mimic PLK1 recruitment to L2, and analyzed their ability to associate with mitotic chromatin. Notably, both WT and SSTP mutant L2-GFP-PLK1 localized to mitotic chromatin in prometaphase-arrested cells to a similar extent (Fig. 6B). These results suggested that indeed CDK1 phosphorylation of the SSTP site was primarily important to recruit PLK1.

### CDK1 and PLK1-L2 phosphosites are crucial to establish a persistent HPV infection

Finally, establishing long-term persistent infections is critical for the transformation of infected cells. The importance of CDK1- and PLK1-mediated L2 phosphorylations for HPV genome persistence was investigated by infection of primary keratinocytes with HPV16 WT, SSTP212AAAA, and T265A quasiviruses containing a recombinant HPV18 genome with a selectable marker^74^. Importantly, persistent infection with these genomes can be blocked by neutralizing antibodies, demonstrating that canonical infection is critical for persistence of this recombinant HPV genomes^74^. As for HPV16 PsV infection, transformation of keratinocytes was completely abrogated, when cells were infected with HPV16 SSTP212AAAA quasiviruses, whereas HPV16 T265A exhibited no colonies for two different keratinocyte donors and drastically reduced colonies for an additional donor (Figs. 6C, D) highlighting the importance of CDK1 and PLK1 for HPV16 infection and subsequent persistence.

## Discussion

In this work, we delineate how nuclear delivery of viral genomes through tethering to mitotic chromatin is mechanistically and functionally regulated by master mitotic kinases for HPVs. Our work not only identified novel phospho-motifs within the HPV minor capsid protein L2, but also that sequential CDK1- and PLK1-mediated phosphorylation of these motifs during mitosis regulated this vital step in the virus entry process in a timely fashion (Fig. 6E).

The HPV16 capsid protein L2 is known to be highly post-translationally modified^63^, however, the roles of these modifications remain largely elusive. Here, we demonstrate that phosphorylation at SSTP 212–215 was mediated by CDK1, whereas PLK1 functionally phosphorylated L2 at residue T265 upon CDK1 priming. This is reminiscent of cellular CDK1 priming events that render cellular targets substrates for PLK1^56,75,76^. The SSTP motif primed by CDK1 enabled PDB-mediated binding of PLK1 to L2 in vitro and in vivo indicating that viral protein and cellular kinase interact as a kinase-substrate pair. Based on L2 mutants, PLK1 inhibition, and the use of an L2-PLK1 fusion protein, sequential L2 phosphorylation by CDK1 and PLK1 was crucial for tethering the viral genome to mitotic chromatin thereby enabling nuclear delivery.

Virus entry occurs as stepwise procedure regulated by sequential cellular cues such as receptor binding for endocytosis or exposure to endosomal low pH for uncoating^77^. CDK1- and PLK1-mediated phosphorylations of L2, likely occurring at the Golgi as for other cellular proteins^78,79^, would provide such a cue to promote transition from the Golgi apparatus to mitotic chromatin during G_2_/M transition. In addition, L2 interaction with and phosphorylation by the two master mitotic kinases is likely to trigger a specific L2 conformation, or to generate a new interface able to interact with factors residing on mitotic chromatin for tethering.

Our work indicated that several additional L2 phosphorylations occured in vivo and in vitro. Their potential importance for nuclear delivery remains will have to be studied by identifying the sites, their regulating kinases, and potentially phosphatases. While they may be regulated by kinases other than CDK1 and PLK1 or unimportant for nuclear delivery, it is tempting to speculate that they provide extra charges to create a synergistic high-affinity interaction motif on L2 or to fulfill independent functions similar to cellular PLK1 substrates such as haspin or the kinesin Kif2b, respectively^80,81^, in particular given the leaky nature of the T265A mutant for vDNA delivery and subsequent persistent infection.

It is plausible that HPVs evolved to hijack this mechanism to ensure timely activation of viral transit from Golgi to chromatin. Moreover, since L2 as mostly intrinsically disordered protein needs to interact with various cellular partners during virus transit from the plasma membrane to mitotic chromatin^82^, appropriately timed phosphorylation would indeed provide an elegant trigger to enable a switch of virus-host interactions promoting a post-Golgi step in the entry program. Our work provided evidence that this step involves tethering of viral genomes to mitotic chromatin through L2. However, L2 phosphorylation may also influence the transit of the Golgi-derived vesicle harboring the virus from the cytosol to mitotic chromatin. Eventually, dephosphorylation of L2 after cytokinesis may be another cue in the entry program perhaps for releasing L2 from tethering to chromatin.

PLK1 has been attributed to both pro- and antiviral roles in previous studies^83–86^. These known roles, however, are linked to viral gene expression or replication but not nuclear delivery of viral genomes. Viruses entering the nucleus during mitosis all possess a dedicated viral protein mediating chromatin tethering, e.g. prototype foamy virus Gag p68 and murine leukemia virus Gag pp12. Thus, it may be that also gamma-type retroviruses delivering their genomes during mitosis into the nucleus employ CDK1 and PLK1-mediated phosphorylations for a similar purpose as observed here. Previous work indeed provides the first evidence that PLK1 is ultimately involved in the integration of the foamy virus genome into the host DNA^87^. Thus, these two rather unrelated families of viruses, the *Papillomaviridae* and the *Retroviridae* may rely on similar mechanisms for the nuclear delivery of genetic information during mitosis.

## Methods

All the reagents and resources used in this study are detailed in Supplementary Table 2.

### Cell culture

HeLa, HEK293, and HEK293T cells were from ATCC. HaCaT cells originated from N. Fusenig (DKFZ, Heidelberg, Germany)^84^. HeLa H2B-mCherry cells were a kind gift of Daniel Gerlich^88^. HEK293TT cells were a kind gift of J.T. Schiller (NIH, Bethesda, USA). Cells were cultured in DMEM (Sigma) supplemented with 10% Fetal Bovine Serum (Capricorn) at 37 °C and 5% CO_2_. HeLa H2B-mCherry cells were additionally supplemented with 500 µg/ml G418 (Capricorn). HeLa L2-EGFP/H2B-mCherry cells were generated by transfection of HeLa H2B-mCherry cells with pL2-EGFP plasmid, followed by clonal selection using 125 µg/mL Hygromycin B. Cells were regularly tested for mycoplasma contamination.

### Transfections

Transfection of cells was performed one day after seeding at a confluency of 50–60% using Lipofectamine 2000 (ThermoFisher) according to the manufacturer’s instructions.

### Pharmacological treatments and cell cycle synchronization

For pharmacological inhibition of PLK1, BI2536 (Selleckchem), BI6727 (Volasertib, Selleckchem), and poloxin (Selleckchem) were added to cells at the indicated final concentrations in growth medium. For inhibition of Aurora A kinase, Alisertib (MLN8237, Selleckchem) was added to cells at the indicated final concentrations in growth medium. Arrest of cells in S-phase and mitosis was achieved by supplementing growth medium with 3 µM aphidicolin (Sigma) or 330 nM nocodazole (Sigma), respectively, for 16 h. Arrest of cells at the G_2_/M boundary was achieved by growth medium with 9 µM RO-3306 (Selleckchem) for 20 h. Release from cell cycle arrest was performed by three washes with pre-heated (37 °C) growth medium.

### Plasmids generation

pSheLL L2-WT, pSheLL L2-SSTP212AAAA, pSheLL L2-S213A, pSheLL L2-T214A, pSheLL L2-T265A, pSheLL BPV1-L2-WT, -TSTP239AAAA, T241A, pSheLL HPV18-L2-WT, -SSTP211AAAA, -T213A, pXULL L2-BirA WT, and pXULL L2-BirA SSTP212AAAA have been generated by using the QuikChange II XL Site-Directed Mutagenesis Kit (Agilent), according to manufacturer’s instructions. All mutants were verified by Sanger sequencing (Eurofins). HPV16 pL2-EGFP SSTP212AAAA, -S212A, -S213A, -T214A, -P215A, BPV1 pL2-EGFP TSTP239AAAA, -T241A, HPV18 pL2-EGFP SSTP211AAAA, -T213A, and HPV5 pL2-EGFP T249A were generated from pL2-EGFP by site-directed mutagenesis using the QuikChange II XL Site-Directed Mutagenesis Kit (Agilent Technologies) according to manufacturer’s instruction. For immunoprecipitations, HPV16 pL2-3xHA SSTP212AAAA, S212A, S213A, T214A, and P215A were generated from pL2-3xHA WT^4^ with mutagenic primers by using the QuikChange II XL Site-Directed Mutagenesis Kit (Agilent Technologies) according to manufacturer’s instruction. pcDNA3-Plk1(829) was a gift from Jonathon Pines (Addgene plasmid #39845). pcDNA3 PBD wt (Nigg AH12) was a gift from Erich Nigg (Addgene plasmid #41162). 3xFLAG-PLK1 was generated by cloning a 3xFLAG sequence into pcDNA3-Plk1(829). 3xFLAG-PLK1 K82R, T210D, and H538A/K540M mutants were generated with mutagenic primers by using the QuikChange II XL Site-Directed Mutagenesis Kit (Agilent Technologies) according to the manufacturer’s instruction. All mutants were verified by Sanger sequencing (Eurofins). All primers used are listed in Supplementary Table 2.

### Virus preparation

HPV16 PsVs HPV16-L2(WT), HPV16-L2(SSTP212AAAA), HPV16-L2(S213A), HPV16-L2(T214A), HPV16-L2(T265A), BPV1 PsVs BPV1-L2(WT/TSTP239AAAA, T241A), HPV18 PsVs HPV18-L2(WT, SSTP211AAAA, T213A), HPV16-L2-BirA(WT) and HPV16-L2-BirA (SSTP212AAAA) containing reporter plasmids (pGL3 for HPV16-L2-BirA viruses or pClneo GFP for all the other viruses) or 5-ethynyl-2′-deoxyuridine (EdU)-labeled DNA was performed were prepared according to standard procedures^89–91^. Briefly, HEK293TT were co-transfected with the according to pSheLL or pXULL and pGL3 or pCIneo, and cells were harvested 48 h post transfection. In the case of EdU-labeling, EdU 6 h post-transfection was added to a final concentration of 20 µM. After virion maturation for 24 h in a medium containing 0.35% Brij58, 25 mM ammonium sulfate, and benzonase (0.25%), HPV16 PsVs were purified either by OptiPREP gradients (for pSheLL generated virions and EdU) or CsCl gradients (for pXULL generated virions) and ultracentrifugation.

### Virus characterization

Mutant HPV16 PsVs were characterized by electron microscopy. Purified PsVs were diluted in virion buffer to a concentration of 1 × 10^8^ particles/μl. PsVs were transferred on a Formvar-coated and carbon-spottered copper grid and sedimented for 10 min. Negative staining was performed by incubating sedimented PsVs with 1% phosphotungstic acid (pH 7.0) for 7 min and letting to dry. Samples were analyzed at 80 kV on a FEI-Tecnai 12 electron microscope (FEI, Eindhoven, Netherlands). Images were acquired with an Olympus Veleta 4k CCD camera.

### Infectivity assays

About 5 × 10^4^ HeLa or HaCaT cells were seeded in 12-well plates 1 day prior to infection with HPV16, BPV1, or HPV18. In order to obtain ~20% infected cells at 48 h p.i., comparable wild type and mutant L2 were used to infect cells (25–50 ng L1) and high virus amounts (250–500 ng L1 capsid protein) were employed to detect any residual infectivity for the mutant virus. The inoculum was exchanged at 2 h p.i. with fresh medium. Cells were fixed 48 h p.i. in 4% PFA, and infectivity was assessed by flow cytometry (Beckman Coulters Galioos) for GFP expression.

### L2-BirA penetration assays

About 6 × 10^5^ HaCaT-GFP-BAP cells were seeded in 24-well plates. The following day, cells were infected with 600, 200, or 67 ng L1/well of HPV16 WT or SSTP212AAAA L2-BirA PsVs. 20 h p.i., samples were rinsed with PBS, surface virus was removed by an alkaline PBS (pH10.6) wash followed by another PBS rinse, and samples were processed for reducing SDS-PAGE followed by Western blot analysis^40^. L2 was detected by mouse monoclonal anti-L2 (K4) (a kind gift of Martin Müller) and GFP by rabbit anti-GFP (Takara Bio Clonetech). IR-Dye conjugated secondary antibodies anti-mouse and anti-rabbit (LI-COR Biosciences GmbH) were used and blots were imaged on the LI-COR Odyssey Infrared Imaging System.

For inhibition studies, the viral inoculum (2 × 10^8^ viral genome equivalents/well) was not removed from cells, and luciferase assays were performed at 24 h p.i. DMSO carrier or PLK1 inhibitors BI2536 and BI6727 (each at 100 nM final concentration) were added at the time of infection and left for the duration.

### L2 chromosomal association assay

Recruitment of HPV16, BPV1, or HPV18 WT and mutant L2-EGFP to mitotic chromatin was investigated^4^. For this, 5 × 10^3^ HeLa H2B-mCherry was seeded on optical 96-well plate one day prior to transfection. Cells were transfected with 100 ng pL2-EGFP constructs using Lipofectamine 2000. Cells were treated 24 h post transfection with 330 nM nocodazole for 16 h to enrich for mitotic events. Cells were fixed in 4% PFA for 15 min, washed thrice, and permeabilized in 0.1% PBS/TritonX-100 for 15 min. After three washes with PBS, the actin cytoskeleton was stained with phalloidin Atto 647 (Sigma) to highlight the cell cortex, required for downstream segmentation and analysis. To study the effects of PLK1 inhibition on L2 recruitment to mitotic chromatin, 8 × 10^3^ HeLa L2-EGFP/H2B-mCherry were seeded on optical 96-well plate and let adhere for 24 h, in absence of G418 (Capricorn) and Hygromycin B (Capricorn). After 24 h, cells were treated with nocodazole and inhibitors as described in the pharmacological treatment section. Cells were fixed, permeabilized, and stained as above. Medial confocal planes were acquired using a Zeiss Axio Observer.Z1 spinning disk microscope (Visitron Systems), with a ×40 PlanApo oil immersion objective.

### vDNA localization studies

To study the effects of PLK1 inhibition on vDNA delivery from incoming virions, 5 × 10^4^ HeLa cells were seeded on glass coverslips in 12-well plates one day prior to infection and synchronization. Cells were infected with 50 ng L1/well EdU-labeled HPV16 PsVs^4^. Two hours p.i., the inoculum was exchanged with fresh growth medium supplemented with 9 µM RO-3306 for 20 h to enrich a G_2_/M cell population. About 20 h post synchronization, cells were released by three rapid washes in pre-heated (37 °C) growth medium, and subsequently incubated in growth medium with nocodazole or nocodazole/BI2536 at indicated concentrations. Cells were fixed 30 min after release in mitosis. Cells were permeabilized in 0.5% PBS/TritonX-100. The EdU-labeled vDNA was detected using the EdU-Click-IT Alexa Fluor 488 reaction Kit (Invitrogen) according to the manufacturer’s instructions. Golgi cis- and medial cisternae were detected using anti-Giantin antibody (BioLegend #PRB-114C) and mitotic chromatin was stained with Hoechst 33258 (Sigma). Confocal slices were acquired using a Zeiss LSM800 confocal microscope with a ×63 PlanApo oil immersion objective. Intensity-based colocalization analysis and quantitation were performed using IMARIS (Bitplane).

### Phosphorylation assays

#### Phos-Tag™ phosphorylation assay

To assess the phosphorylation state of HPV16-L2 wild type and SSTP mutant, 1 × 10^5^ HeLa or HEK293 cells were seeded in six-well plate. Cells were transfected with 1 µg of plasmid DNA. About 24 h post transfection, cells were treated with aphidicolin or nocodazole to arrest cell cycle in S-phase or mitosis, respectively, for additional 18 h. Cells were lysed in RIPA buffer and total protein amount was quantified using the BCA Protein Assay Kit (Thermo Fisher) according to the manufacturer’s instructions. Protein lysates (25 µg) were analyzed by reducing SDS-PAGE and immunoblotting. SDS-PA gels were supplemented with 50 µM Phos-Tag™ (Wako Chemicals) and 100 µM MnCl_2_ (Sigma), according to manufacturer’s instruction.

L2 was detected with mouse anti L2 sc-65709 (Santa Cruz) and IR-Dye conjugated secondary antibody anti-mouse (LI-COR Biosciences GmbH) were used. Blots were imaged by LI-COR Odyssey Infrared Imaging System (LI-COR Biosciences GmbH) or Intas ChemostarTouch (INTAS Science Imaging Instruments GmbH).

#### in vitro kinase assays

To study the phosphorylation kinetics of HPV16-L2 in vitro, purified unfolded WT and SSTP212AAAA mutant L2 were refolded^92^. For this, bacterially expressed 6xHis-L2 was diluted to 3 mg/mL and reduced with DTT. Subsequently, buffer exchange was performed by dialysis into refolding buffer (0.5 M L-Arginine, 10 mM Tris, 200 mM NaCl and 5% glycerol, pH 7.5) at 4 °C for 16 h. Following centrifugation at 24,000 × *g* at 4 °C for 8 h, refolded L2 amount was quantified by Coomassie Brilliant Blue staining using a standard bovine albumin curve. Purified 6xHis-PLK1 and the CDK1-CyclinB:CKS1 complex were kindly provided by Marion Pesenti (Musacchio Lab)^57,93^. A molar kinase-to-substrate ratio of 1:50 was adopted for all reactions using 100 nM of PLK1 and/or CDK1-CyclinB:CKS1 and 5 µM of WT or SSTP212AAAA mutant L2. Reactions were started by adding 2 mM ATP and incubated for 16 h at 4 °C in kinase buffer (0.5 M L-Arginine, 10 mM Tris, 200 mM NaCl, 1 mM TCEP, 10 mM MgCl_2,_ pH 75 at 4 °C). The reactions were stopped by adding 5× sample buffer (250 mM Tris, 10% SDS, 30% glycerol, 20 mM DTT 0.05% Bromophenol Blue, pH 6.8) and incubation at 95 °C for 10 min.

### Immunoprecipitation

To study interaction of L2 with PLK1, protein immunoprecipitation (IP) followed by western blot analysis was performed. 5 × 10^6^ HEK293 cells were seeded on 100 mm dishes and transfected with 20 µg plasmid pL2-3xHA WT or SSTP212AAAA mutant constructs. For co-transfection of pL2-3xHA, p3xFLAG-PLK1, pVps26B-myc and pPBD-myc constructs, 10 µg of each construct were transfected. 24 h post transfection, cells were treated with aphidicolin, nocodazole, or nocodazole and BI2536 for additional 16 h. Cells were lysed in IP lysis buffer (150 mM NaCl, 1% IGEPAL CA-630, 50 mM Tris, 50 mM NaF, pH 7.4) supplemented with protease inhibitor cocktail (Sigma), PhosSTOP (Sigma), 10 mM MgCl_2_ and 1000 unit benzonase (Millipore), for 30 min at 4 °C in an overhead rotator. Lysates were centrifuged for 20 min with 12000 × *g* at 4 degrees and pre-clearing was performed with Protein G Agarose (Sigma). Incubation with primary antibodies (HA.11, 1:150; FLAG M2 1:200, c-myc 1:200) was performed for 16 h at 4 °C. Protein G Agarose beads (Sigma) were added for 4 h, and IP was performed according to manufacturer’s instructions. Immunoprecipitated L2 was eluted with 5× Sample Loading buffer (250 mM Tris, 10% SDS, 30% glycerol, 20 mM DTT 0,05% Bromophenol Blue, pH 6.8) and boiling samples at 95 °C for 10 min. Samples were analyzed by SDS-PAGE and western blot.

### Mass spectrometry

To identify the phosphorylation residues on HPV16-L2 protein IP followed by tandem mass spectrometry was performed. About 1.5 × 10^7^ HEK293 cells were seeded on 150 mm dishes and transfected with 100 µg pL2-3xHA or pL2-3xHA SSTP212AAAA. About 24 h post transfection, cells were treated with 3 µM aphidicolin, 330 nM nocodazole, or 330 nM nocodazole, and 100 nM BI2536 for additional 16 h. Cell were lysed in IP lysis buffer (150 mM NaCl, 1% IGEPAL CA-630, 50 mM Tris, 50 mM NaF, pH 7.4) supplemented with protease inhibitor cocktail (Sigma), PhosSTOP (Sigma), 10 mM MgCl_2_, and 1000 unit benzonase (Millipore), for 30 min at 4 °C in an overhead rotator. Lysates were centrifuged for 20 min with 12,000 × *g* at 4 °C and pre-clearing was performed with Protein G Agarose (Sigma). Incubation with primary antibody (HA.11, 1:150) was performed for 16 h at 4 °C. Protein G Agarose beads (Sigma) were added for 4 h and IP was performed according to the manufacturer’s instructions. Immunoprecipitated L2 was eluted with elution buffer (25 mM Tris, 4% SDS, pH 7.4), and prepared for mass spectrometry. Eluted L2 protein as well as bacterially produced L2 protein that was used as substrate in the in vitro kinase assay (see above) were first subjected to acetone precipitation. IP samples were dissolved in 8M urea, reduced by the addition of DTT (20 mM; 1 h, 37 °C), and alkylated using iodoacetamide (55 mM; 30 min at RT in darkness), while the L2 from the in vitro kinase assay was resuspended in 50 mM ammonium bicarbonate buffer and directly alkylated using chloroacetamide (55 mM; 30 min at RT in the dark). Subsequent to dilution (IP) and the addition of CaCl_2_ (10 mM final concentration) digestion was initiated by the addition of chymotrypsin (0.5–1 µg; dissolved in 1 mM HCl) and incubated for 16 h at 25 °C. The digests were quenched by the addition of TFA to a final concentration of 1% and subsequently desalted using C18 Stage tips.

For mass spectrometry analysis, samples were eluted from the Stage tips twice using 20 µl of Buffer B (80% acetonitrile, 0.5% formic acid) dried in a Eppendorf evaporator and dissolved in 10 µl Buffer A (0.5% formic acid in water) each. LC-MSMS was performed using a Q Exactive HF mass spectrometer (Thermo Fisher Scientific) online coupled to an EASY nLC 1200 nano-HPLC via a self-packed fused silica emitter column (360 µm OD × 75 µm ID × 25 cm L; Nanoseparations, NL) that had been pressure filled with 1.9 µm C18 beads (Reprosil pur, C18-AQ, Dr. Maisch; Ammerbuch, Germany). The column was placed in a PRSO column oven mounted on top of the NanoSpray Flex source (both Thermo Fisher Scientific) with the column oven temperature set to 45 °C.

Immunoprecipitation samples were online separated using a multilinear gradient running from 2–20% Buffer B in 150 min, from 20–50% B in 90 min, and from 50–90% B in 10 min with a flow rate of 250 nl/min. The column was flushed with 90% B for additional 5 min before re-equilibration at starting conditions (Buffer A). In contrast, samples from the in vitro kinase assay were separated using a linear gradient running from 3–35% Buffer B in 75 min at a flow rate of 300 nl/min. The column was also flushed with Buffer B for 5 min before returning to starting conditions.

The Orbitrap HF mass spectrometer was operated in positive mode, alternating in data-dependent mode between survey scans in the orbitrap (mass range m/z = 300–1750; resolution *R* = 60000; target value = 3E6; max IT 100 ms) and MS/MS acquisition (HCD) of the 17 most intense ion peaks detected (resolution *R* = 15.000; target value = 1E5; max IT = 50 msec; NCE = 27). Spectra were recorded all in profile mode. Dynamic exclusion of already identified spectra was allowed and set to 20 s. Mass spectrometry settings for the analysis of the L2 protein from in vitro kinase samples were similar, except for adaptations to the scan range (m/z = 300–1650), the max IT (50 ms), and the NCE (NCE = 28).

Raw MS data were processed using MaxQuant (v. 1.6.6.0 (IP) or v. 1.6.17.0 (in vitro kinase assay)) with the built-in Andromeda search engine. Tandem mass spectra obtained from the measurement of the immunoprecipitated samples were searched against the human papillomavirus as well as the human UniProtKB database (UP000009251_333760.fasta; UP000005640_9606.fasta; versions from 04/2019), while spectra obtained from the measurement of the in vitro phosphorylated, bacterially expressed L2 protein were searched against the sequence of the L2 protein proper (L2_capsid.fasta) as well as against the uniprotKB database for *E.coli* (UP000000625_83333.fasta, version from 04/2019). In both instances, databases were concatenated with reversed sequence versions of all entries, supplemented also with common lab contaminants. Carbamidomethylation on cysteine residues was set as fixed modification for the search, while oxidation at methionine, deamidation of asparagine residues, acetylation of protein N-termini and phosphorylation on serine, threonine, and tyrosine were set as variable modifications. Chymotrypsin was defined as the digesting enzyme, allowing a maximum of two missed cleavages and requiring a minimum length of 6 or 7 amino acids. The maximum allowed mass deviation was 20 ppm for MS and 0.5 Da for MS/MS scans. The match between run function was enabled as well as the calculation of iBAQ intensity values. Protein groups were regarded as being unequivocally identified with a false discovery rate (FDR) of 1% for both the peptide and protein identifications. Phosphorylation sites were accepted when they were identified with a localization probability of >0.75 (class I sites).

### Gaussia protein complementation assay

For the GPCA (Gaussia Protein Complementation Assay), 2.5 × 10^4^ HEK293T cells were seeded in 96-well plates. After 24 h, cells were transfected with 100 ng of pSPICA-N2 (encoding G2 fragment of Gaussia princeps luciferase), pSPICA-N2-HPV16-L2, or pSPICA-N2-HPV5 L2 or pSPICA-N2-BPV1-L2 and 100 ng of pSPICA-N1 (encoding G1 fragment of Gaussia princeps luciferase) or pSPICA-N1-PLK1wt, pSPICA-N1-PLK1 T210D, pSPICA-N1-PML, pSPICA-N1-HPV16 L1, pSPICA-N1-IRF3, pSPICA-N1-E6AP. At 24 h post transfection, cells were washed with PBS and lysed with Renilla Lysis Buffer (Promega). Gaussia princeps luciferase enzymatic activity was measured using a Berthold Centro LB960 luminometer by injecting coelenterazine (Promega) and counting luminescence for 10 s. Results were expressed as normalized relative luminescence (RLUC). For a given proteins pair A/B, RLUC = (G1−A + G2−B)/[(G1−A + G2) + (G1 + G2−B)]^70^.

### Quantitative colony formation assay

400,000 HFKs were plated on 10 cm dishes containing terminally irradiated NIH-J2 fibroblasts. The next day, NIH-J2 fibroblasts were removed using 0.48 mM EDTA in PBS, and cells were infected using 150 vge/cell. The cell number was based on counts for an extra plate grown under identical conditions. 16 h post infection 1E6 terminally irradiated NIH-J2 fibroblasts were added to the infected cells. The next day (i.e., 2 days post infection, cells were treated with 200 µg/ml Neomycin (G418 disulfate, cat. #108321-42-2). Media was replaced with fresh media containing 200 µg/ml Neomycin until macroscopic colonies were visible on the wildtype virus-infected plates. At this point, fibroblasts were removed using 0.48 mM EDTA in PBS, and HFK colonies were stained using 5 mg/ml MTT (3-(4, 5-dimethylthiazol-2-yl)-2, 5-diphenyltetrazolium bromide) solution in F-media. Cells are stained for 4 h at 37 °C at which point colonies are visualized and photographed. Individual colonies are enumerated and plotted. Note that variability in colony formation between different genetic donors has been described^74^.

### Quantitation and statistical analysis

#### Quantitation of HPV16 infectivity

Quantitation of HPV16-infected HeLa and HaCaT cells was performed by scoring GFP-positive cells 48 h p.i. by FACS. For each condition, 1 × 10^5^ events (cells) were scored, for three to four independent experiments. Data were plotted with GraphPad Prism 7. Statistical analysis was performed using a Student’s *t* test^94–100^.

#### Quantitation of L2 chromosomal association assay

L2 chromosomal association was calculated using CellProfiler^4^. The chromosomal association index (CAI) was quantified and plotted with GraphPad Prism 7. Statistical analysis was performed using a Student’s *t* test.

#### Quantitation of vDNA chromosomal tethering during HPV16 infection

Quantitation of HPV16-EdU-labeled vDNA was performed using IMARIS Coloc function (Bitplane). The total amount of vDNA residing on mitotic chromatin or the Golgi apparatus was determined by colocalization of vDNA with mitotic chromatin signal (Hoechst) or Golgi signal (Giantin). Coloc signal was quantified and plotted with GraphPad Prism 7. Statistical analysis was performed using a Student’s *t* test.

### Reporting summary

Further information on research design is available in the Nature Portfolio Reporting Summary linked to this article.

## Supplementary information

Supplementary Information

Reporting Summary

## Data Availability

The mass spectrometry data generated in this study have been deposited in the PRIDE database under accession code PXD028704. Supplementary information Source data are provided with this paper.

## Source data

Source Data
