# Supplementary material for: Master mitotic kinases regulate viral genome delivery during papillomavirus cell entry
Source: Nat Commun. 2023 Jan 23;14:355. doi: 10.1038/s41467-023-35874-w (PMC9868124; doi:10.1038/s41467-023-35874-w)
Supplement: Supplementary file 1 — Supplementary Information [file 41467_2023_35874_MOESM1_ESM.pdf]

Supplementary information:

Master mitotic kinases regulate viral genome delivery during papillomavirus cell entry

Matteo Rizzato[1], Fuxiang Mao[1], Florian Chardon[1], Kun-Yi Lai[1, 2], Ruth Villalonga-Planells[1], Hannes C. A. Drexler[3], Marion E. Pesenti[4], Mert Fiskin[5], Nora Roos[6], Kelly M. King[7], Shuaizhi Li[8], Eduardo R. Gamez[8#], Lilo Greune[9], Petra Dersch[9], Claudia Simon[6], Murielle Masson[5], Koenraad Van Doorslaer[7,10], Samuel K. Campos[8], and Mario Schelhaas[1, 2]

1) Institute of Cellular Virology, Westphalian Wilhelms-University of Münster, Münster, Germany
2) Interfaculty Centre ‘Cells in Motion’ (CiM), Westphalian Wilhelms-University of Münster, Münster, Germany
3) Max Planck Institute for Molecular Biomedicine, Münster, Germany
4) Max Planck Institute of Molecular Physiology, Dortmund, Germany
5) UMR 7242 Biotechnologie et signalisation cellulaire, CNRS, UdS, ESBS, Illkirch, France
6) Institute of Medical Virology and Epidemiology of Viral Diseases, Tübingen, Germany
7) School of Animal and Comparative Biomedical Sciences, University of Arizona, Tucson, Arizona, USA
8) Department of Immunobiology, University of Arizona, Tucson (AZ), U.S.A.
9) Institute of Infectiology, Westphalian Wilhelms-University of Münster, Münster, Germany
10) Cancer Biology Graduate Interdisciplinary Program, Genetics Graduate Interdisciplinary Program, UA Cancer Center, University of Arizona Tucson, Arizona, USA
#Current affiliation: Department of Tropical Medicine, Medical Microbiology and Pharmacology, University of Hawai’i at Manoa, Honolulu, Hawaii 96813-5525

*Corresponding author: schelhaas@uni-muenster.de

		PLK1	CDK1	CDK1 +PLK1	Localizationprobability	Score
T	209			O	0.94	180.41
S	213		O	O	0.64	162.09
T	214		O	O	0.99	203.75
T	244		O	O	0.78	174.26
T	245		O	O	0.99	182
T	247		O	O	0.39	88.017
T	265			O	0.91	71.221
S	274	O			0.94	54.19
T	295		O	O	0.99	102.26
S	304	O			1.00	70.942
T	314		O		0.49	66.809
S	316		O		0.49	66.809
S	319			O	0.99	157.33

Supplementary Table 1. Listed are phosphorylations on L2 WT after in vitro phosphorylation of purified L2 after incubation with purified PLK1, CDK1, or PLK1 and CDK1 as identified by mass spectrometry analysis. Orange indicates phosphorylation of sites exclusively in the presence of both kinases.

Key resource table

REAGENT or RESOURCE	SOURCE	IDENTIFIER (Cat.)
**Antibodies**		
IRDye 800CW donkey anti rabbit (1:5000)	LI-COR Biosciences GmbH	#P/N 925-32213
IRDye 800CW goat anti mouse (1:5000)	LI-COR Biosciences GmbH	#P/N 925-32210
Mouse monoclonal anti Cdc2 p34 (CDK1) (1:1000)	Santa Cruz Biotechnology	#sc-54
Mouse monoclonal anti c-myc (9E10) (1:1000)	ThermoFisher	#13-2500
Mouse monoclonal anti FLAG M2 (1:1000 for WB, 1:150 for IP)	Sigma-Aldrich	#F1804
Mouse monoclonal anti HA.11 (1:1000 for WB, 1:150 for IP)	BioLegend (previously Covance)	#901516
Mouse monoclonal anti HPV16 L2 (1:2000)	Santa Cruz Biotechnolgy	#sc-65709
Mouse monoclonal anti L2 (K4L2(20-38)) (1:1000)	Martin Müller, DKFZ	[88]
Mouse monoclonal anti PLK1 [36-298] (1:1000)	Abcam	#ab17057
Rabbit monoclonal anti TCTP (D10F2) (1:1000)	Cell Signaling	#5128
Rabbit polyclonal anti GAPDH (1:5000)	Proteintech	#10494-1-AP
Rabbit polyclonal anti GFP (1:2000)	Takara Bio Clontech	#632592
Rabbit polyclonal anti Giantin (1:200)	BioLegend	#PRB-114C
Rabbit monoclonal anti pSTP (1:1000)	Cell Signaling	D73F6 #5243
Rabbit polyclonal anti phospho-TCTP (pSer46) (1:1000)	Cell Signaling	#5251
**Bacterial and Virus Strain**		
E. coli BL21 DE3	ThermoFisher	#EC0114
E. coli strain ZYCY10P3S2T	System Biosciences	MN900A-1
p16sheLL	[12]	N/A
**Chemicals and Recombinant Proteins**		
Adenine	Sigma	A-2786
Alkaline Phosphatase	New England Biolabs	#M0290S
Arabinose	Sigma	W325501

REAGENT or RESOURCE	SOURCE	IDENTIFIER (Cat.)
Aphidicolin from *N. sphaerica*	Sigma	#A0781
ATP	PJK GmbH	#102262
Benzonase	Millipore	#1016950001
BI2536	Selleckchem	#S1109
BI6727 (Volasertib)	Selleckchem	#S2235
Cholera toxin	Calbiochem	227036
Coelentrazine	Promega	#E289A
Coomassie Brilliant Blue G250	Serva	#17524.01
6xHis-CDK1-CyclinB:CKS1 (CCC)	Andrea Musacchio	[89]
D-Glucose	Sigma	G7021
Deoxycholic Acid	Serva	#18330.02
DMEM	Sigma	#D5796
DMSO	Sigma	#D2438-50ML
EDTA	Sigma	#E9984
Epidermal growth factor	Invitrogen	PHG0311
Fetal Bovine Serum	Capricorn	#FBS-11A
G418 (Geneticin)	Capricorn	#G418-R
Glycerol	Roth	#3783.1
Ham's F-12	Invitrogen	11765-062
Hoechst 33258	Sigma	#94403
High-glucose Dulbecco's modified Eagle's medium (DMEM)	Invitrogen	11960-044
Hydrocortisone	Sigma	H-4001
Hygromycin B	Capricorn	#Hyg-H
IGEPAL CA-630	Sigma	#I8896
Insulin	Sigma	I1882

REAGENT or RESOURCE	SOURCE	IDENTIFIER (Cat.)
L-Arginine	Sigma	#A5131
Lipofectamine 2000	ThermoFisher	#10696153
$MgCl_2$	Sigma	#M8266
$MnCl_2$	Sigma	#M5005
Alisertib (MLN8237)	Selleckchem	#S1133
MTT (3-(4,5-dimethylthiazol-2-yl)-2,5-diphenyltetrazolium bromide	Research Products International	M92050-5.0
NaF	Roth	#P756.1
Neomycin	Invitrogen	108321-42-2
Nocodazole	Sigma	#M1404
OptiMEM	Invitrogen	#11058-021
Optiprep	Sigma	#D1556
PFA	Sigma	#P6148
Phalloidin Atto 647	Sigma	#65906
PhosSTOP	Sigma	#04906837001
Phos-Tag™	Wako Chemicals	#304-93521
Phusion HF DNA Pol	New England Biolabs	#M0530S
6xHis-PLK1	Andrea Musacchio	[57]
Poloxin	Selleckchem	#S5893
Polyethyleneimine, linear, MW 25000	Santa Cruz Biotechnology	Sc-360988
Protease Inhibitor	Sigma	#11836170001
Protein G Agarose	Sigma	#11243233001
Refolded L2 SSTP212AAAA mutant	[90]	N/A
Refolded L2 WT	[90]	N/A
Renilla Lysis Buffer	Promega	#E291A
RO-3306	Selleckchem	#S7747

REAGENT or RESOURCE	SOURCE	IDENTIFIER (Cat.)
T4 Ligase	New England Biolabs	#M0202S
TCEP	ThermoFisher	#20490
Triton X-100	Sigma	#X100
Trypsin-EDTA	Sigma	#T3924
y-27632	Chemdea	CD0141
**Critical Commercial Assays**		
BCA Protein Assay Kit	Thermo Fisher	#23225
EdU-Click-IT Reaction Kit<br>Maxi-prep kit	Life Technologies<br>Macherey-Nagel	#C10337<br>#740414100
PCR cleanup kit	Qiagen	#28106
QuikChange II XL Kit	Agilent	#1020305
**Experimental Models: Cell Lines**		
Human foreskin derived keratinocytes (HFK) strain 14	This study	N/A
Human foreskin derived keratinocytes (HFK) strain 44	This study	N/A
Human foreskin derived keratinocytes (HFK) strain 6	This study	N/A
Human: HaCaT	[91]	N/A
Human: HEK293	ATCC	#CRL-1573
Human: HEK293T	ATCC	#
Human: HEK293tt	Christopher Buck	N/A
Human: HeLa	ATCC	#CCL-2
Human: HeLa L2-EGFP/H2B-Cherry	[43]	N/A
G418-resistant NIH-3T3 J2 murine fibroblasts	[92]	N/A
**Recombinant DNA**		
p3xFLAG-PLK1	This study	N/A
p3xFLAG-PLK1 H538A/K540M	This study	N/A

REAGENT or RESOURCE	SOURCE	IDENTIFIER (Cat.)
p3xFLAG-PLK1 K82R	This study	N/A
p3xFLAG-PLK1 T210D	This study	N/A
p3xFLAG-Ran WT	[43]	N/A
pL2-3xHA P215A	This study	N/A
pL2-3xHA S212A	This study	N/A
pL2-3xHA S213A	This study	N/A
pL2-3xHA SSTP212AAAA	This study	N/A
pL2-3xHA T214A	This study	N/A
pL2-3xHA WT	[4]	N/A
pCIneo-GFP	[93]	N/A
pL2-EGFP P215A	This study	N/A
pL2-EGFP S213A	This study	N/A
pL2-EGFP S319A	This study	N/A
pL2-EGFP SSTP212AAAA	This study	N/A
pL2-EGFP T209A	This study	N/A
pL2-EGFP T214A	This study	N/A
pL2-EGFP T265A	This study	N/A
pL2-EGFP WT	[5]	N/A
pL2-EGFP-PLK1 SSTP212AAAA	This study	N/A
pL2-EGFP-PLK1 WT	This study	N/A
pL2-EGFP WT (HPV18)	This study	N/A
pL2-EGFP SSTP211AAAA (HPV18)	This study	N/A
pL2-EGFP T213A (HPV18)	This study	N/A
pL2-EGFP WT (BPV1)	This study	N/A
pL2-EGFP TSTP239AAAA (BPV1)	This study	N/A

REAGENT or RESOURCE	SOURCE	IDENTIFIER (Cat.)
pL2-EGFP T241A (BPV1)	This study	N/A
pL2-EGFP WT (HPV5)	This study	N/A
pL2-EGFP T249A (HPV5)	This study	N/A
p16sheLL L2-WT	[93]	N/A
p16sheLL L2-SSTP212AAAA	This study	N/A
p16sheLL L2-S213A	This study	N/A
p16sheLL L2-T214A	This study	N/A
p16SheLL L2-T265A	This study	N/A
p18SheLL L2-WT	This study	N/A
p18SheLL L2-SSTP211AAAA	This study	N/A
p18SheLL L2-T213A	This study	N/A
BPV1 pSheLL L2-WT	This study	N/A
BPV1 pSheLL L2-TSTP239AAAA	This study	N/A
BPV1 pSheLL L2-T241A	This study	N/A
HPV18-Neo genome cloned into pMC.BESBX	This study	N/A
pMC.BESBX	System Biosciences	MN100B-1
pMEP4-HPV18 E1	PMC5120138	N/A
pMEP9-HPV18 E2	PMC5120138	N/A
pXULL-L2-BirA WT	[40]	N/A
pXULL-L2-BirA SSTP212AAAA	This study	N/A
pGL3	[94]	N/A
pPBD-myc	[95]	N/A
pcDNA3-PLK1	[96]	Addgene_39845
pVps26B-myc	[97]	N/A
pSPICA-N1	[98]	N/A

REAGENT or RESOURCE	SOURCE	IDENTIFIER (Cat.)
pSPICA-N2	[98]	N/A
pSPICA-N2-HPV5 L2	This study	N/A
pSPICA-N2-BPV1 L2	This study	N/A
pSPICA-N2-HPV16 L2	This study	N/A
pSPICA-N1-PLK1wt	This study	N/A
pSPICA-N1-PLK1 T210D	This study	N/A
pSPICA-N1-PML	This study	N/A
pSPICA-N1-HPV16 L1	This study	N/A
pSPICA-N1-IRF3	This study	N/A
pSPICA-N1-E6AP	This study	N/A
**Softwares and Algorithms**		
Bitplane IMARIS version 9	Bitplane	http://www.bitplane.com/
CellProfiler version 4.0.6	Broad Institute	https://cellprofiler.org/
Fiji release 2017 May 30	NIH	https://fiji.sc/
FlowJo version 8	FLOWJO, LCC	https://www.flowjo.com/
ImageStudio Lite version 5.0	LI-COR Biosciences GmbH	https://www.licor.com/bio/image-studio-lite/
Prism version 7	GraphPad	https://www.graphpad.com/scientific-software/prism/
Affinity Designer version 1.10.1	Serif Europe Ltd	https://affinity.serif.com/en-gb/designer/
**Primers**	**sequence**	
HPV16 L2 SSTP/AAAA Rv	acCCGAACACCGTTACCgcCgcCgCCgCGATTCCGGGTAGCCGTc	
HPV16 L2 SSTP/AAAA Fw	GACGGCTACCCGGAATCGCGGCGGCGGCGGTAACGGTGTTCGGGT	
HPV16 L2 S212A Fw	CCCAACACCGTGACCGCCAGCACCCCCATCCCC	
HPV16 L2 S212A Rv	GGGGATGGGGGTGCTGGCGGTCACGGTGTTGGG	
HPV16 L2 S213A Fw	CCCAACACCGTGACCAGCGCCACCCCCATCCCC	
HPV16 L2 S213A Rv	GGGGATGGGGGTGGCGCTGGTCACGGTGTTGGG	

Primers	sequence
HPV16 L2 T214A Fw	CCCAACACCGTGACCAGCAGCGCCCCCATCCCC
HPV16 L2 T214A Rv	GGGGATGGGGGCGCTGCTGGTCACGGTGTTGGG
HPV16 L2 P215A Fw	ACCGTGACCAGCAGCACCGCCATCCCCGGCAGC
HPV16 L2 P215A Rv	GCTGCCGGGGATGGCGGTGCTGCTGGTCACGGT
HPV16 L2 T265A Fw	TATCGACGTGGATAACgCCCTGTATTTCAGCAGCAA
HPV16 L2 T265A Rv	TTGCTGCTGAAATACAGGGCGTTATCCACGTCGATA
HPV18 L2 SSTP211AAAA Fw	GAAGAGCCAATCGCCGCCGCCGCCCTCCCCACCGT C
HPV18 L2 SSTPP211AAAA rv	GACGGTGGGGAGGGCGGCGGCGGCGATTGGCTC TTC
HPV18 L2 T213A Fw	GAAGAGCCAATCtCCtCCGCCcCCCTCCCCACCGTC
HPV18 L2 T213A Rv	GACGGTGGGGAGGGgGGCGGaGGaGATTGGCTCT TC
HPV18 L2 T262A Fw	GAACCCGTCGATACCgCCCTGACCTTCGACC
HPV18 L2 T262A rv	GGTCGAAGGTCAGGGcGGTATCGACGGGTTC
HPV5 L2 SSTP AAAA Fw	CCACCGAGGAGGgCAgCCgCCgCCCTGCCGAGGAA CCAG
HPV5 L2 SSTP AAAA Rv	CTGGTTCCTCGGCAGGGCGGCGGCTGCCCTCCTC GGTGG
HPV5 L2 T249A Fw	CCACCGAGGAGGTCATCCgCCCCCCTGCCGAGGAA CCAG
HPV5 L2 T249A Rv	CGTTCTCGAAGATGTTGGCGACCTCCTCTTCG
HPV5 L2 T303A Fw	CGAAGAGGAGGTCgCCAACATCTTCGAGAACG
HPV5 L2 T303A Rv	CGTTCTCGAAGATGTTGGCGACCTCCTCTTCG
BPV1 L2 T241A Fw	CCCCCCGCACCAGCgCCCCCCGCTCCATC
BPV1 L2 T241A Rv	GATGGAGCGGGGGGCGCTGGTGCGGGGGG
BPV1 L2 TSTP239AAAA Fw	GGCTCCCCCCGCGCCGCCGCCGCCCGCTCCATCG CC
BPV1 TSTP239AAAA rv	GGCGATGGAGCGGGCGGCGGCGGCGCGGGGGG AGCC

Supplementary Table 2. List of the reagents and resources used in this study.

Supplementary Figure 1.

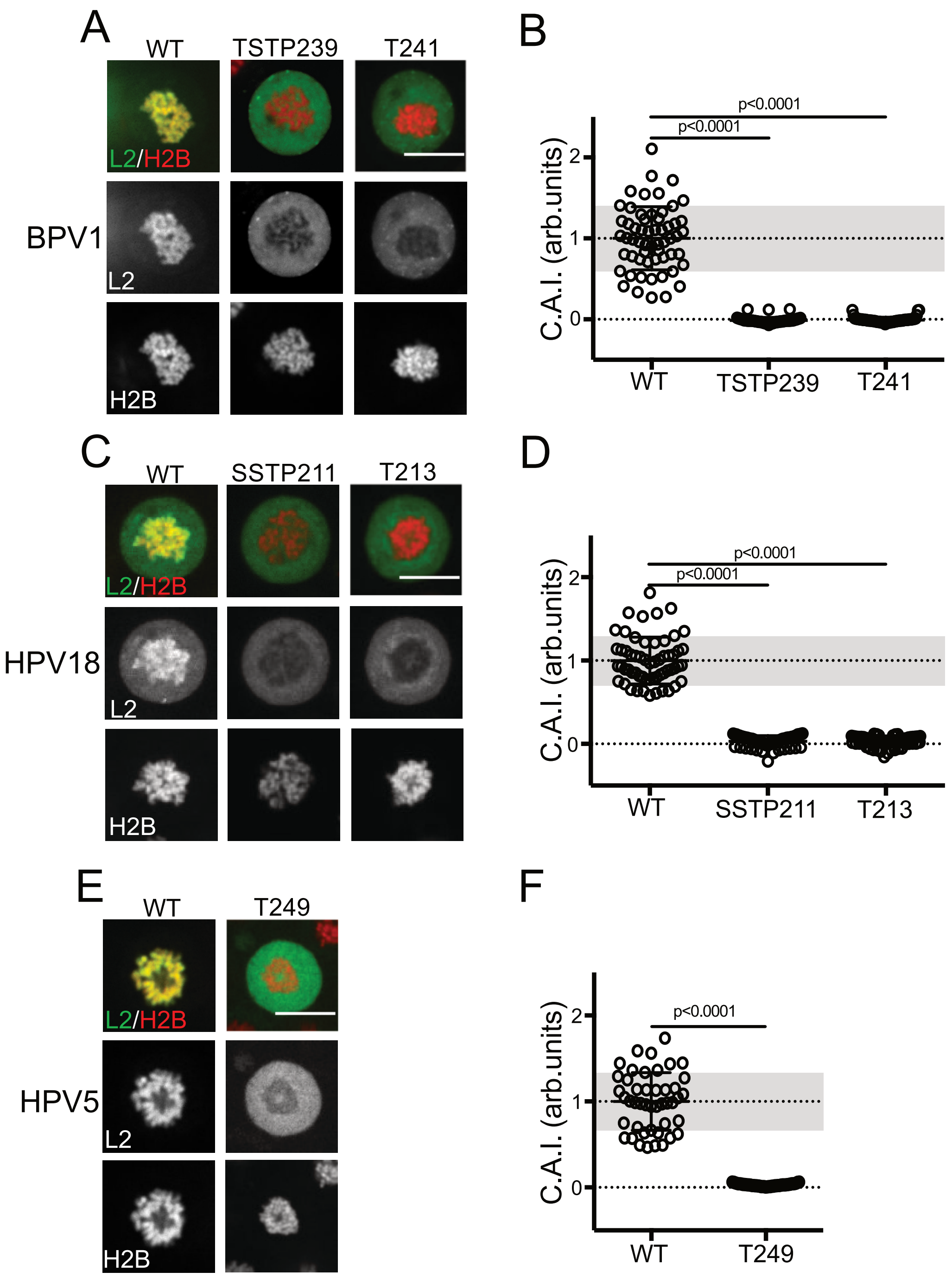

Supplementary figure 1. Chromosomal association assay of ectopically expressed wild type and mutant L2-EGFPs. (A) BPV1, (C) HPV18 and (E) HPV5 in HeLa H2B-mCherry cells during mitosis; images display representative single medial planes of spinning disk confocal microscopy with L2-EGFP in green and H2B-mCherry in red as indicated. Scale bars 10 µm. Quantification of (B) BPV1, (D) HPV18 and (F) HPV5 display the chromosomal association index (CAI) of individual cells (circles). 50 cells from three independent experiments were analyzed. Displayed is the average of three independent experiments ± SD. Statistical significance was assessed by two-tailed Student’s t-test to wildtype (WT). Source data are provided as a Source Data file.

Supplementary Figure 2.

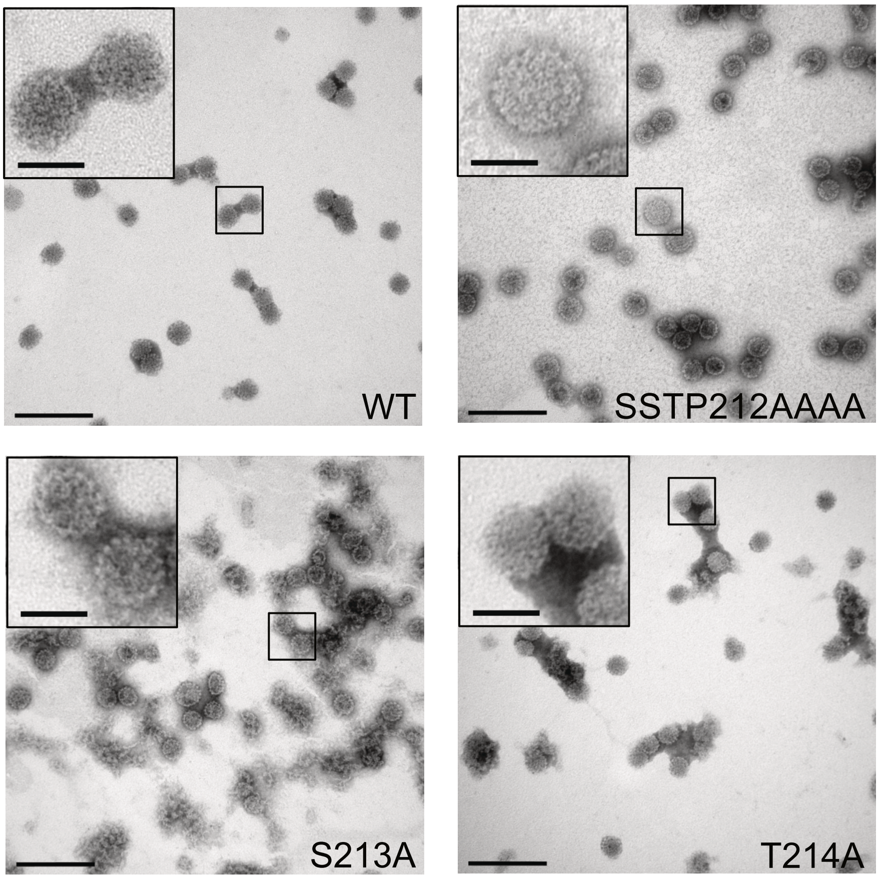

Supplementary figure 2. Electron microscopy of HPV16WT and L2 mutant PsVs. (A) Purified L2 WT, SSTP212AAAA, S213A and T214A mutant HPV16 PsVs were purified as described in methods and processed for and imaged by electron microscopy. Depicted are representative images of single virions of three different virus preparations. Scale bars: 50 nm.

Supplementary Figure 3.

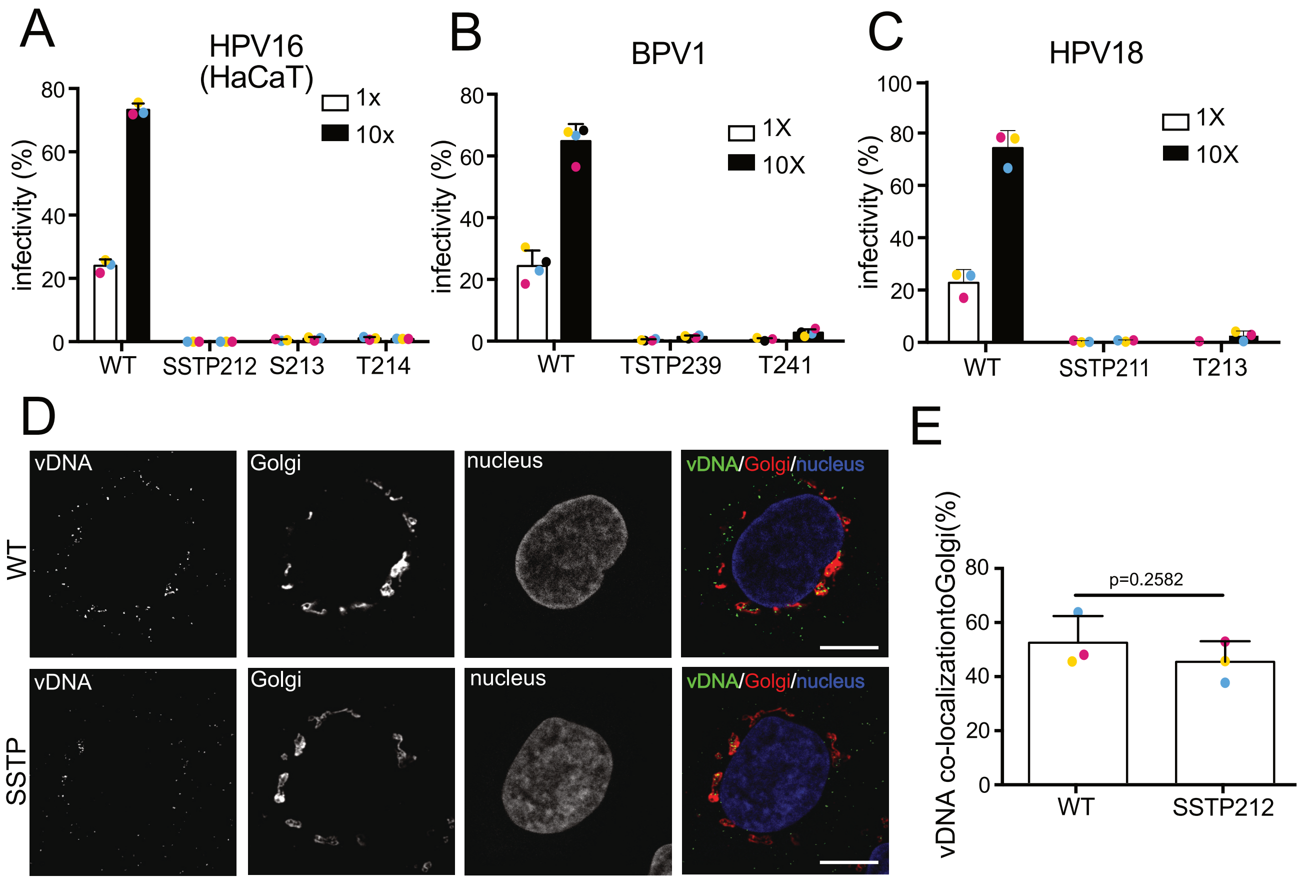

Supplementary figure 3. Infectivity and Golgi delivery of HPV WT and L2 mutants. (A) HaCaT cells were infected with L2 WT and mutant HPV16 PsVs as indicated. Infectivity was assessed 48 hours post infection (p.i.) by scoring GFP positive cells by flow cytometry. Virus amounts correspond to 1x = 50 ng L1, 10x = 500 ng L1. Displayed is the average of three independent experiments ± SD. (B) HeLa cells were infected with L2 WT and mutant BPV1 PsVs as indicated. Virus amounts correspond to 1x = 15 ng L1, 10x = 150 ng L1. Displayed is the average of four independent experiments ± SD. (C) HeLa cells were infected with HPV18 L2 WT and mutant PsVs as indicated. Virus amounts correspond to 1x = 20 ng L1, 10x = 200 ng L1. Displayed is the average of three independent experiments ± SD. (D) HeLa cells were infected with WT and SSTP212 L2 HPV16-EdU PsV for 24 h. Displayed are representative medial confocal slices of the subcellular localization of vDNA (EdU, green), Golgi (Giantin, red) and nuclei (Hoechst, blue) in interphase cells. Scale bars: 5 µm. (E) Quantification of (D) for three independent experiments with eight cells/experiment. The overlap of vDNA/Golgi was quantified using intensity based colocalization analysis (IMARIS Coloc function). Displayed is the average of three independent experiments ± SD. For all quantifications, statistical significance was assessed by two-tailed Student's t-test to wildtype (WT). Source data are provided as a Source Data file.

Supplementary Figure 4.

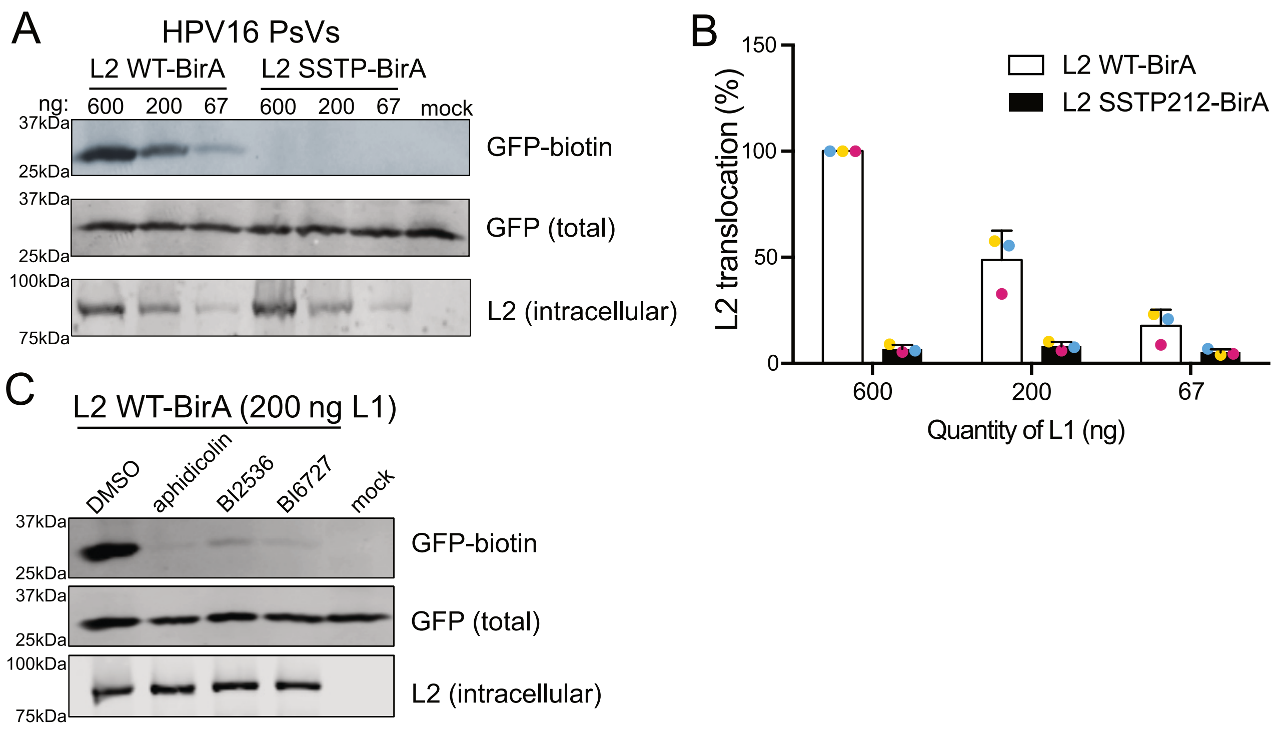

Supplementary figure 4. HPV16 WT and L2 mutant BirA assay. (A) HPV16 WT and SSTP212AAAA L2-BirA membrane penetration assay representing L2 accessibility to the cytosol from the limiting membrane, as described in materials and methods and in[40]. HeLa cells were infected with the indicated amounts of WT or SSTP212 L2-BirA HPV16 PsVs for 20 h. Depicted is a representative example from Western blot analysis against GFP (Takara Bio Clontech #632592) and against intracellular L2 (anti L2 K4, Martin Müller). (B) Quantification by densitometry of (A), for three independent experiments ± SD. (C) HPV16 WT L2-BirA membrane penetration assay in presence of aphidicolin (3 µM), BI2536 (100 nM), BI6727 (100 nM) as in Supplementary Figure 4A, assessed by Western blot analysis. Depicted is a representative example from Western blot analysis against GFP (Takara Bio Clontech #632592) and against intracellular L2 (anti L2 K4, Martin Müller). Source data are provided as a Source Data file.

Supplementary Figure 5.

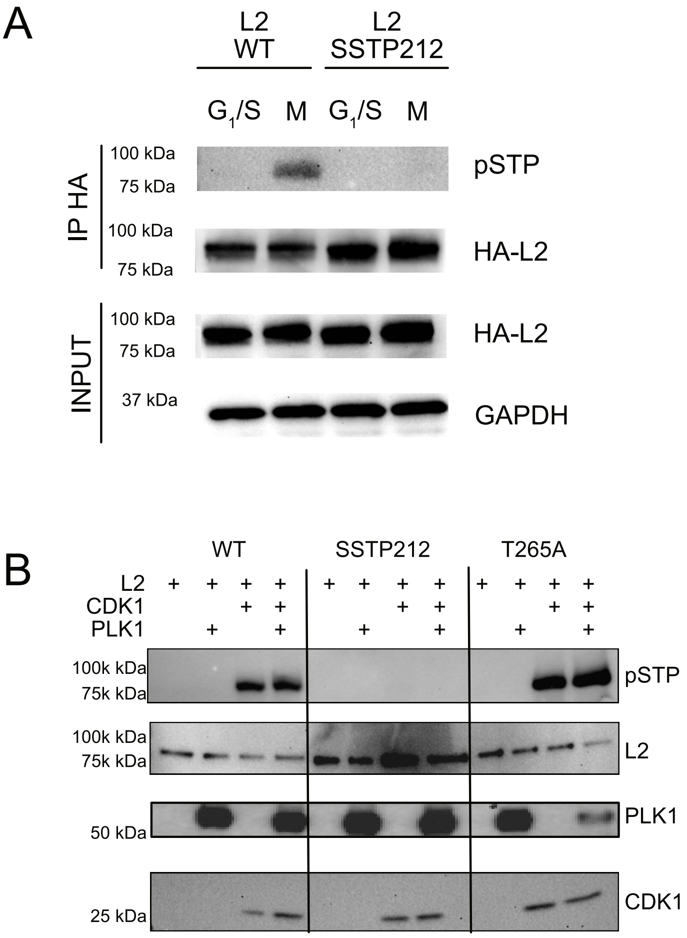

Supplementary figure 5. STP phosphorylation of L2. (A) HEK293 cells were transfected with a WT L2-3xHA or L2-SSTP212AAAA expression construct. Subsequently cells were arrested in G_1/S-phase, or in mitosis by aphidicolin or nocodazole treatment as described for Figure 2A.L2 was immunoprecipitated using antibody against HA tag (BioLegend #901516), from cell lysates (input), and analyzed by Western blotting with antibodies against HA tag (BioLegend #901516), pSTP (Cell Signaling 5243), or GAPDH (Proteintech #10494-1-AP). Depicted are representative examples of three independent experiments. (B) In vitro kinase assay of WT, SSTP212AAAA and T265A purified, refolded 6xHis-L2 in presence of 6xHis-PLK1, or of 6xHis-CDK1-CyclinB:CKS1 (CCC) complex, or of both kinases, was performed as described in methods. Depicted are representative examples from Western blot analysis against pSTP (Cell Signaling 5243), L2 (Santa Cruz sc-65709), PLK1 (Abcam ab17057) and CDK1 (Santa Cruz #sc- 54) of SDS-PAGE of the in vitro kinase assay in presence of ATP of three independent experiments. Source data are provided as a Source Data file.

Supplementary Figure 6.

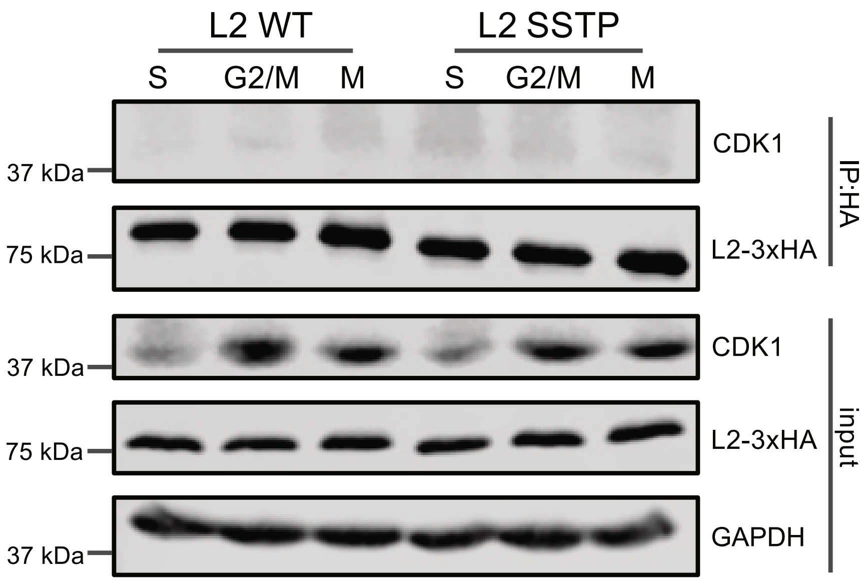

Supplementary figure 6. CDK1 immunoprecipitation with ectopically expressed L2. (A) HEK293 cells were transfected with WT and mutant L2-3xHA expression constructs, and subsequently arrested in S-phase, M-phase, or at the G2/M border using aphidicolin (3 µM), nocodazole (330 nM) or RO3306 (9 µM). Depicted is a representative example of three independent experiments from Western blot analysis against HA tag (BioLegend #901516), CDK1 (Santa Cruz sc-54), or GAPDH (Proteintech #10494-1-AP) as loading control of SDS-PA gel electrophoresis of endogenous CDK1 immunoprecipitates (Santa Cruz sc-54). Source data are provided as a Source Data file.

Supplementary Figure 7.

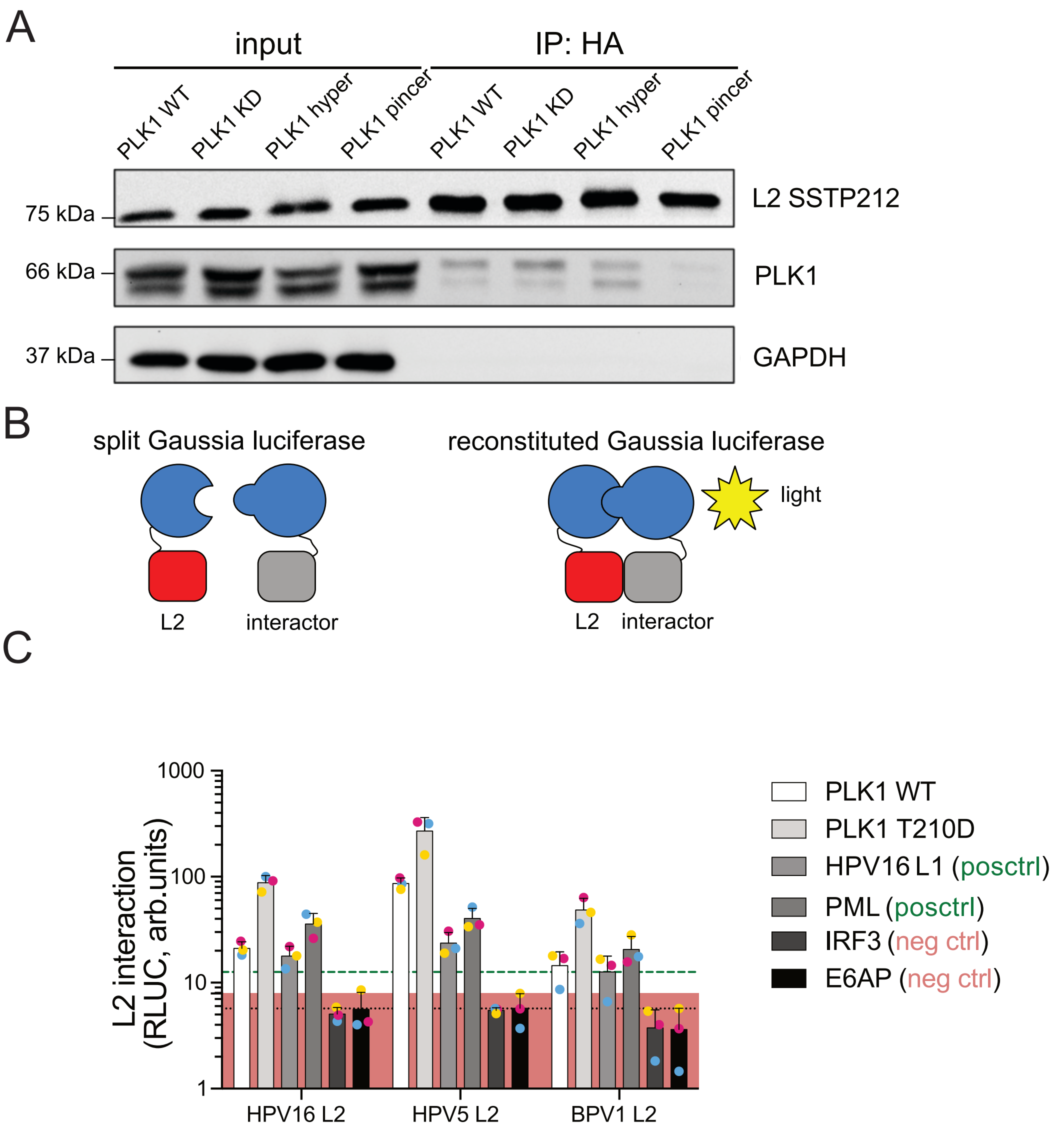

Supplementary figure 7. L2 interactions with PLK1 mutants. (A) HPV16 L2 SSTP control for Figure 3E. HEK293 cells were co-transfected with pL2-3xHA SSTP212AAAA and p3xFLAG-PLK1 mutants(WT:wildtype,KD:kinasedeadK82R,hyper:hyperactivatedT210D,pincer:Polo-box domains dead, H538A K540M). Depicted is a representative example from Western blot analysis against L2 (Santa Cruz sc-65709), against FLAG tag for 3xFLAG-PLK1 (Sigma-Aldrich #F1804), against GAPDH (Proteintech #10494-1-AP) and of SDS-PA gel electrophoresis of SSTP212AAAA L2-3xHA (Santa Cruz sc-65709) and 3xFLAG-PLK1 (Sigma-Aldrich #F1804), immunoprecipitates. Representative blots for two to three independent experiments are shown. (B) Schematic depiction of the Gaussia Protein Complementation assay, performed as described in materials and methods and in[70]. (C) Quantification of L2 interaction with WT and hyperactivated (T210D) PLK1 for HPV16, HPV5 and BPV1 L2. As positive control, L1 and PML were used, as they are known positive interactors of L2, whereas E6AP and IRF3 were used as negative controls. Quantification was shown as the average of three independent experiments ± SD. Source data are provided as a Source Data file.

Supplementary figure 8.

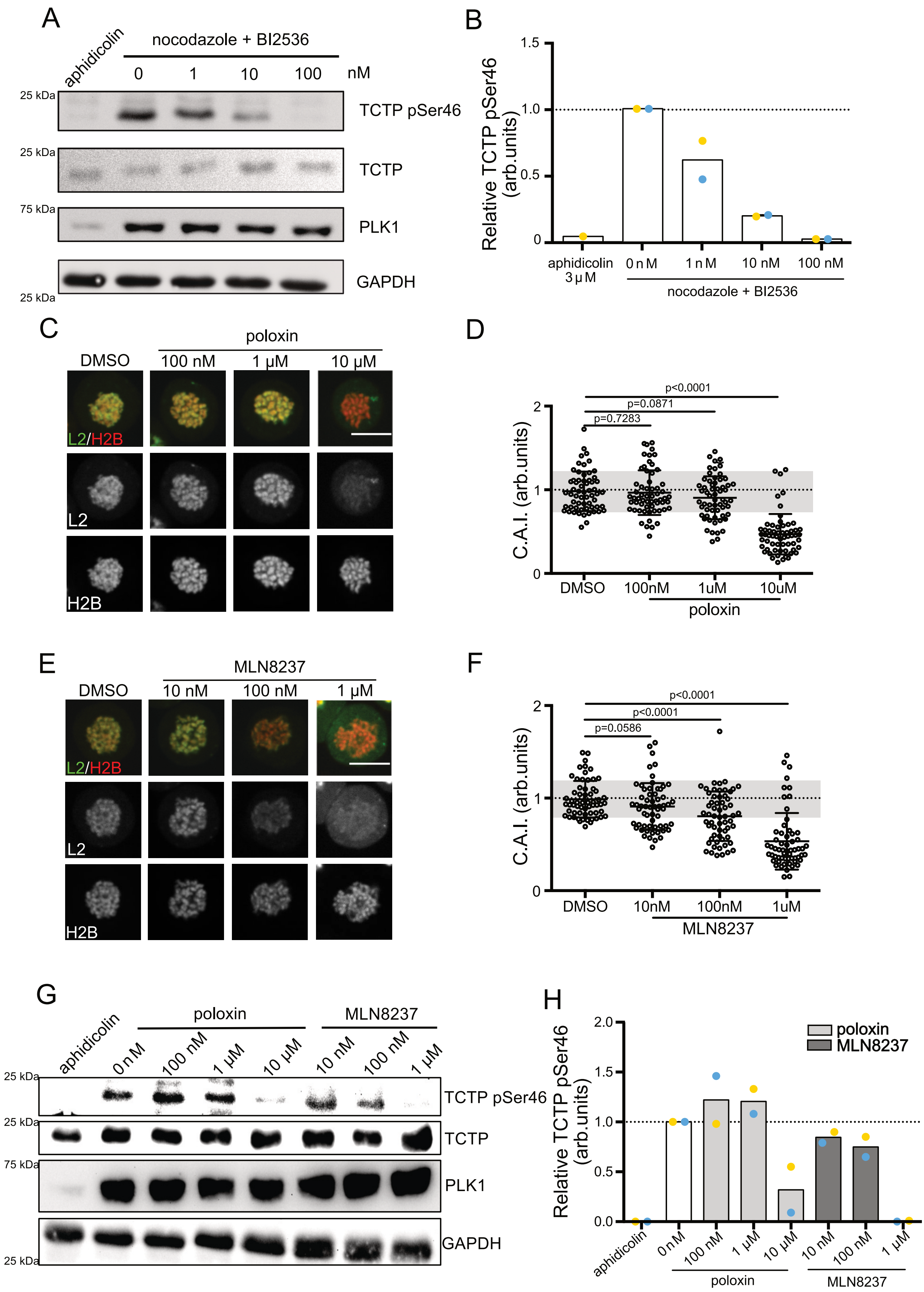

Supplementary figure 8. PLK1 inhibition studies. (A) Western blot analysis of PLK1 inhibition in presence of BI2536, assessed by phospho-Ser 46 levels of TCTP, a validated PLK1 cellular target during mitosis. HeLa cells were arrested in S-phase by aphidicolin (3 µM) or M-phase by nocodazole (330 nM) and BI2536 (1-100 nM) treatment. Cell lysates were harvested at indicated time points after block release and subjected to Western blot analysis. Depicted is a representative example from Western blot analysis against TCTP pSer46 (Cell Signaling #5251), TCTP (Cell Signaling #5128), PLK1 (Abcam ab17057) and against GAPDH (Proteintech #10494-1-AP) (B) Quantification by densitometry of (A), for two independent experiments. (C, E) HeLa cells stably expressing L2-GFP/H2B-mCherry were incubated in presence of nocodazole (330 nM) to arrest cells in mitosis, and either poloxin (10 µM, C) or MLN8237 (1 µM, E) for 16 hours. Subsequently, cells were processed for chromosomal association assay. Depicted are representative single medial planes of spinning disk confocal microscopy with L2-EGFP in green and H2B-mCherry in red as indicated. Scale bars 10 µm. (D) and (F) Quantification of (C) and (E), respectively, displaying the chromosomal association index (CAI) of individual cells (circles). 50 cells from three independent experiments were analyzed. Displayed is the average of three independent experiments ± SD. (G) as (A) in the presence of poloxin or MLN8237. (H) Quantification by densitometry of (G), for two independent experiments. Statistical significance was assessed by two-tailed Student's t-test to control. Source data are provided as a Source Data file.

Supplementary Figure 9.

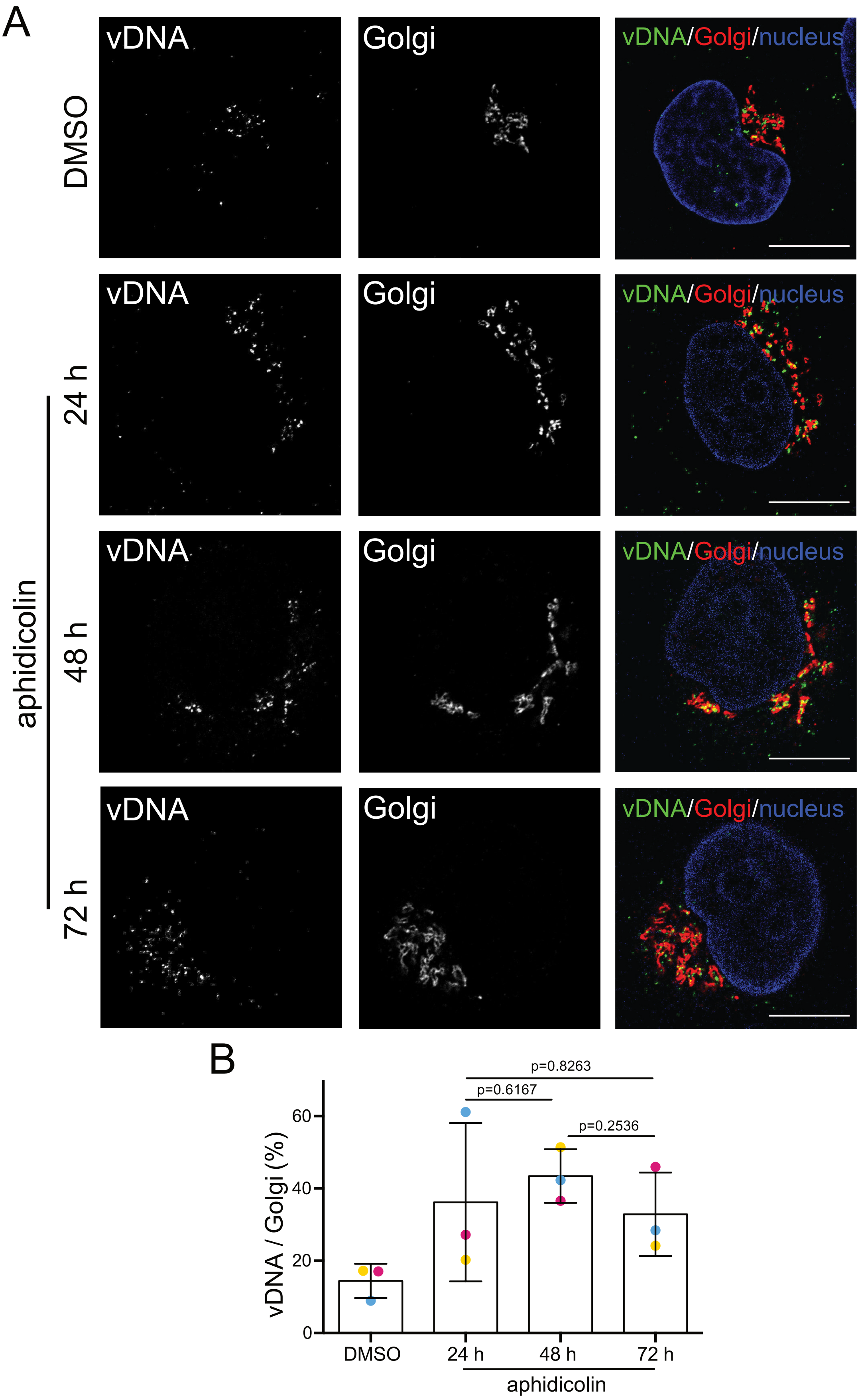

Supplementaryfigure 9. Retainment of vDNAat the Golgi. (A) HeLa cells were infected with WT L2 HPV16-EdU PsV in the presence of aphidicolin (3 µM) treatment for 24, 48, and 72 h, respectively. DMSO served as solvent control (24 h). Displayed are representative medial confocal slices of the subcellular localization of vDNA (EdU, green), Golgi apparatus (Giantin, red) and nuclei (Hoechst, blue). Scale bar: 5 µm. Quantification of (B) vDNA colocalization with Giantin was assessed by 60 cells from three independent experiments with 20 cells/experiment. The overlap of vDNA/Golgi was quantified using intensity-based colocalization analysis (IMARIS Coloc function). Displayed is the average of three independent experiments ± SD. Statistical significance was assessed by two-tailed Student's t-test to control. Source data are provided as a Source Data file.
