# Supplementary material for: Master mitotic kinases regulate viral genome delivery during papillomavirus cell entry
Source: Nat Commun. 2023 Jan 23;14:355. doi: 10.1038/s41467-023-35874-w (PMC9868124; doi:10.1038/s41467-023-35874-w)
Supplement: Supplementary file 2 — Reporting Summary [file 41467_2023_35874_MOESM2_ESM.pdf]

nature portfolio

Corresponding author(s): Mario Schelhaas

Last updated by author(s): Dec 14, 2022

Reporting Summary

Nature Portfolio wishes to improve the reproducibility of the work that we publish. This form provides structure for consistency and transparency in reporting. For further information on Nature Portfolio policies, see our Editorial Policies and the Editorial Policy Checklist.

Statistics

For all statistical analyses, confirm that the following items are present in the figure legend, table legend, main text, or Methods section.

n/a	Confirmed	
☐	☒	The exact sample size (n) for each experimental group/condition, given as a discrete number and unit of measurement
☐	☒	A statement on whether measurements were taken from distinct samples or whether the same sample was measured repeatedly
☐	☒	The statistical test(s) used AND whether they are one- or two-sided<br>*Only common tests should be described solely by name; describe more complex techniques in the Methods section.*
☐	☒	A description of all covariates tested
☒	☐	A description of any assumptions or corrections, such as tests of normality and adjustment for multiple comparisons
☐	☒	A full description of the statistical parameters including central tendency (e.g. means) or other basic estimates (e.g. regression coefficient) AND variation (e.g. standard deviation) or associated estimates of uncertainty (e.g. confidence intervals)
☐	☒	For null hypothesis testing, the test statistic (e.g. F, t, r) with confidence intervals, effect sizes, degrees of freedom and P value noted<br>*Give P values as exact values whenever suitable.*
☒	☐	For Bayesian analysis, information on the choice of priors and Markov chain Monte Carlo settings
☒	☐	For hierarchical and complex designs, identification of the appropriate level for tests and full reporting of outcomes
☒	☐	Estimates of effect sizes (e.g. Cohen's d, Pearson's r), indicating how they were calculated

Our web collection on statistics for biologists contains articles on many of the points above.

Software and code

Policy information about availability of computer code

Data collection	The following Software has been used for data collection:<br>ImageStudio Lite version 5.0, LI-COR Biosciences GmbH, https://www.licor.com/bio/i<br>mage-studio-lite/
Data analysis	The following software has been used for Data analysis and display:<br>Bitplane IMARIS version 9, Bitplane, http://www.bitplane.com/<br>CellProfiler version 4.0.6, Broad Institute, https://cellprofiler.org/<br>Fiji release 2017 May 30, NIH https://fiji.sc/<br>FlowJo version 8, FLOWJO, LCC https://www.flowjo.com/<br>ImageStudio Lite version 5.0, LI-COR Biosciences GmbH, https://www.licor.com/bio/i<br>mage-studio-lite/<br>Prism version 7, GraphPad, https://www.graphpad.com<br>/scientific-software/prism/<br>Affinity Designer version 1.10.1, Serif Europe Ltd, https://affinity

For manuscripts utilizing custom algorithms or software that are central to the research but not yet described in published literature, software must be made available to editors and reviewers. We strongly encourage code deposition in a community repository (e.g. GitHub). See the Nature Portfolio guidelines for submitting code & software for further information.

Data

Policy information about availability of data

All manuscripts must include a data availability statement. This statement should provide the following information, where applicable:

- Accession codes, unique identifiers, or web links for publicly available datasets
- A description of any restrictions on data availability
- For clinical datasets or third party data, please ensure that the statement adheres to our policy

Source data have been provided with this paper (western blots, and numerical data points for graphs). The mass spectrometry data has been added to PRIDE repository with accession code PXD028704.

Human research participants

Policy information about studies involving human research participants and Sex and Gender in Research.

Reporting on sex and gender	Not applicable, as no human research participants were involved.
Population characteristics	Not applicable
Recruitment	Not applicable
Ethics oversight	Not applicable

Note that full information on the approval of the study protocol must also be provided in the manuscript.

Field-specific reporting

Please select the one below that is the best fit for your research. If you are not sure, read the appropriate sections before making your selection.

[x] Life sciences [ ] Behavioural & social sciences [ ] Ecological, evolutionary & environmental sciences

For a reference copy of the document with all sections, see nature.com/documents/nr-reporting-summary-flat.pdf

Life sciences study design

All studies must disclose on these points even when the disclosure is negative.

Sample size	All sample sizes for infection or microscopy experiments are based on previous work (Aydin I. et al. 2017; Lai K. et al. 2021), which indicates that increasing sample size would not alter the result.
Data exclusions	No data was excluded from this study.
Replication	All experiments unless stated otherwise were replicated at least three times most often with duplicates for each independent replicate.
Randomization	Randomization is not applicable due to the small sample size in Fig. 6C.
Blinding	Blinding occurred for crucial experiments, i.e. for Fig. 1B-D, 2, 4, 5A-D, 6C and Suppl. Fig. 1, 5B, 8C-F.

Reporting for specific materials, systems and methods

We require information from authors about some types of materials, experimental systems and methods used in many studies. Here, indicate whether each material, system or method listed is relevant to your study. If you are not sure if a list item applies to your research, read the appropriate section before selecting a response.

Materials & experimental systems

n/a	Involved in the study
☐	☒ Antibodies
☐	☒ Eukaryotic cell lines
☒	☐ Palaeontology and archaeology
☒	☐ Animals and other organisms
☒	☐ Clinical data
☒	☐ Dual use research of concern

Methods

n/a	Involved in the study
☒	☐ ChIP-seq
☒	☐ Flow cytometry
☒	☐ MRI-based neuroimaging

Antibodies

Antibodies used

The following antibodies were used: IRDye 800CW donkey anti rabbit (#P/N 925-32213, LI-COR Biosciences GmbH, 1:5000), IRDye 800CW goat anti mouse (#P/N 925-32210, LI-COR Biosciences GmbH, 1:5000), Mouse monoclonal anti Cdc p34 (CDK1) (#sc-54, Santa Cruz Biotechnology, 1:1000), Mouse monoclonal anti c-myc (9E10) (#13-2500, ThermoFisher, 1:1000), Mouse monoclonal anti FLAG M2 (#F1804, Sigma-Aldrich, 1:1000 for WB and 1:150 for IP), Mouse monoclonal anti HA. 11 (#901516, BioLegend, 1:1000 for WB and 1:150 for IP), Mouse monoclonal anti HPV16 L2 (#sc-65709, Santa Cruz Biotechnology, 1:2000), Mouse monoclonal anti L2 (K4L2 (20-38)) was a kind gift from Martin Müller, DKFZ. Mouse monoclonal anti PLK1 [36-298] (#ab17057, Abcam, 1:1000), Rabbit monoclonal anti TCTP (D10F2) (#5128, Cell Signaling, 1:1000), Rabbit polyclonal anti GAPDH (#10494-1-AP, Proteintech, 1:5000), Rabbit polyclonal anti GFP (#632592, Takara Bio Clontech, 1:2000), Rabbit polyclonal anti Giantin (#PRB-114C, BioLegend, 1:200 for IF), Rabbit monoclonal anti pSTP (D73F6 #5243, Cell Signaling, 1:1000), Rabbit polyclonal anti phospho-TCTP (pSer46) (#5251, Cell Signaling, 1:1000)

Validation

The information of all antibodies we used can be found in their official website.
IRDye 800CW donkey anti rabbit LI-COR Biosciences GmbH #P/N 925-32213
The antibody was isolated by affinity chromatography using antigens coupled to agarose beads. Based on ELISA, this antibody reacts with the heavy and light chains of rabbit IgG, and with the light chains common to most rabbit immunoglobulins. This antibody was tested by ELISA and/or solid-phase adsorbed to ensure minimal cross-reactivity with bovine, chicken, goat, guinea pig, hamster, horse, human, mouse, rat, and sheep serum proteins, but may cross-react with immunoglobulins from other species. The conjugate has been specifically tested and qualified for Western blot and In-Cell Western™ Assay applications.

IRDye 800CW goat anti mouse LI-COR Biosciences GmbH #P/N 925-32210
Isolation of specific antibodies was accomplished by affinity chromatography using pooled mouse IgG covalently linked to agarose. Based on ELISA and flow cytometry, this antibody reacts with the heavy and light chains of mouse IgG1, IgG2a, IgG2b, and IgG3, and with the light chains of mouse IgM and IgA. This antibody was tested by dot blot and and/or solid-phase adsorbed for minimal cross-reactivity with human, rabbit, goat, rat, and horse serum proteins, but may cross-react with immunoglobulins from other species. The conjugate has been specifically tested and qualified for Western blot applications.

Mouse monoclonal anti Cdc2 p34 (CDK1) Santa Cruz Biotechnology #sc-54
Cdc2 p34 Antibody (17) is an IgG2a κ monoclonal Cdc2 p34 antibody (also designated CDK1 antibody, CDC2/CDK1 antibody or p34 protein kinase antibody) suitable for the detection of the Cdc2 p34 protein of mouse, rat, human and Xenopus laevis origin. Cdc2 p34 Antibody (17) is available as both the non-conjugated anti-Cdc2 p34 antibody form, as well as multiple conjugated forms of anti-Cdc2 p34 antibody, including agarose, HRP, PE, FITC and multiple Alexa Fluor® conjugates. In vertebrates, as in yeast, multiple cyclins have been identified, including a total of eight such regulatory proteins in mammals. In contrast to the situation in yeast, the Cdc2 p34 kinase is not the only catalytic subunit identified in vertebrates that can interact with cyclins. While Cdc2 p34 is essential for the G2 to M transition in vertebrate cells, a second Cdc2-related kinase has also been implicated in cell cycle control. This protein, designated cyclin-dependent kinase 2 (Cdk2) p33, also binds to cyclins and its kinase activity is temporally regulated during the cell cycle. Several additional Cdc2 p34-related cyclin dependent kinases have been identified. These include Cdk3-Cdk8, PCTAIRE-1-3 and KKIALRE.

Mouse monoclonal anti c-myc (9E10) ThermoFisher #13-2500
This Antibody was verified by Relative expression to ensure that the antibody binds to the antigen stated. Antibody specificity was demonstrated by detection of different targets fused to Myc tag in transiently transfected lysates tested. Relative detection of Myc tag was observed across different proteins fused with Myc tag in His-H3-Myc (Lane 3-5) and Myc-p65-V5 (Lane 6), using Anti-Myc Tag Polyclonal Antibody (Product # 13-2500) in Western Blot. This product has been shown to detect Myc Tag at both N- and C- termini of a fusion protein.

Mouse monoclonal anti FLAG M2 Sigma-Aldrich #F1804
The ANTI-FLAG M2 mouse, affinity purified monoclonal antibody binds to fusion proteins containing a FLAG peptide sequence.The antibody recognizes the FLAG peptide sequence at the N-terminus, Met-N-terminus,C-terminus, and internal sites of the fusion protein.

Mouse monoclonal anti HA.11 BioLegend (previously Covance) #901516
This second-generation HA antibody is an excellent substitute for the 12CA5 monoclonal antibody. The HA.11 antibody recognizes the influenza hemagglutinin epitope (YPYDVPDYA) which has been used extensively as a general epitope tag in expression vectors. The extreme specificity of the antibody allows unambiguous identification and quantitative analysis of the tagged protein. The HA.11 antibody recognizes HA epitopes located in the middle of protein sequences as well as at the N- or C-terminus.

Mouse monoclonal anti HPV16 L2 Santa Cruz Biotechnolgy #sc-65709

HPV16 L2 Antibody (2JGmab#5) is a high quality monoclonal HPV16 L2 antibody (also designated human papillomavirus type 16 minor capsid protein L2 antibody, HPV16 L2 antibody, dsDNA alpha papillomavirus 9 (HPV16) L2 protein antibody, or HpV16gp7 (L2) antibody) suitable for the detection of the HPV16 L2 protein of HPV-16 origin. HPV16 L2 Antibody (2JGmab#5) is available as both the non-conjugated anti-HPV16 L2 antibody form, as well as multiple conjugated forms of anti-HPV16 L2 antibody, including agarose, HRP, PE, FITC and multiple Alexa Fluor®conjugates. Human papillomaviruses, particularly type 16 (designated HPV16), infect the genital tract and may lead to cervical cancer. Protection against HPV16 is thought to be provided by neutralizing antibodies directed to the major caspid protein L1 of HPV16. HPV16 L1 forms the pentameric assembly unit of the viral shell, and the binding of HPV16 L1 to the cell surface without the involvement of minor capsid protein L2 is believed to be the first step of HPV16 infection. The L1-binding domain located near the C-terminus of L2 binds L1 prior to completion of capsid assembly and is required for efficient encapsidation of the viral genome. In addition, the C-terminus of L1 is necessary for both DNA binding and DNA packaging. Expression of the late gene L1 is restricted to the upper layers of the infected epithelium. HPV16 L1 is able to package unrelated plasmid DNA in vitro and deliver the foreign DNA to eukaryotic cells with the subsequent expression of the encoded gene. L1 shows a diffuse nuclear distribution whereas L2 is localized to punctate nuclear regions identified as promonocytic leukemia protein oncogenic domains (PODs). Coexpression of L1 and L2 induces a relocalization of L1 into the PODs.

Mouse monoclonal anti L2 (K4L2(20-38)) Martin Müller, DKFZ
Checked in article 'Natural variants in the major neutralizing epitope of human papillomavirus minor capsid protein L2'
Mouse monoclonal anti PLK1 [36-298] Abcam #ab17057
Use a concentration of 1 µg/ml. Detects a band of approximately 66 kDa (predicted molecular weight: 68 kDa).

Rabbit monoclonal anti TCTP (D10F2) Cell Signaling #5128
Western blot analysis of extracts from various cell lines (HeLa, 293, COS-7, C2C12, MTLn3, NIH/3T3) using TCTP (D10F2) Rabbit mAb.

Rabbit polyclonal anti GAPDH Proteintech #10494-1-AP
Various lysates (HEK293, HeLa, Jurkat, NIH3T3, C6, mouse brain, rat brain) were subjected to SDS PAGE followed by western blot with 10494-1-AP (GAPDH antibody) at dilution of 1:20000 incubated at room temperature for 1.5 hours.

Rabbit polyclonal anti GFP Takara Bio Clontech #632592
The Living Colors Full-Length GFP Polyclonal Antibody was raised in a rabbit against recombinant full-length Aequorea coerulescens green fluorescent protein (rAcGFP1). This antibody recognizes native and denatured forms of AcGFP1 and GFP, as well as N- or C-terminal fusion proteins of AcGFP1 expressed in mammalian cells.

Rabbit polyclonal anti Giantin BioLegend #PRB-114C
This antibody is effective in immunoblotting (WB) and immunofluorescence (IF).
*Predicted MW = 367 kD. Western transfer should be conducted in the presence of 0.1% SDS. After transfer, rinse blot with 50% MeOH to remove any residual SDS.

Rabbit monoclonal anti pSTP Cell Signaling D73F6 #5243
Immunoprecipitation of extracts from HeLa cells, untreated or nocodazole-treated (100 ng/ml, 16 hrs), using Phospho-PLK Binding Motif (ST*P) (D73F6) Rabbit mAb (lanes 1 and 2). 10% input is shown in lanes 3 and 4. Western blots were performed using Phospho-PLK Binding Motif (ST*P) (D73F6) Rabbit mAb (upper), RIP (D94C12) XP® Rabbit mAb #3493 (upper middle), MEK1/2 Antibody #9122 (lower middle), and NPM Antibody #3542 (lower).

Rabbit polyclonal anti phospho-TCTP (pSer46) Cell Signaling #5251
Western blot analysis of extracts from U-2 OS cells, untreated or synchronized in mitosis by thymidine block followed by release into nocodazole, using Phospho-TCTP (Ser46) Antibody.

Eukaryotic cell lines

Policy information about cell lines and Sex and Gender in Research

Cell line source(s)	HeLa, HEK293, and HEK293T cells were from ATCC. HaCat cells originated from N. Fusenig (DKFZ, Heidelberg, Germany) and were a kind gift of J.T. Schiller (NIH, Bethesda, USA). HeLa H2B-mCherry cells were a kind gift of Daniel Gerlich. HeLa H2B-mCherry/EGFP-L2 cells were generated in our lab. HEK293TT cells were a kind gift of J.T. Schiller (NIH, Bethesda, USA). HFK and NIH-3T3 J2 cells were a kind gift of Koenraad Van Doorslaer.
Authentication	For daily work, cells were identified by morphologic characteristic. On a regular basis, cell lines were additionally authenticated by DNA barcoding and PCR assays with species-specific primers
Mycoplasma contamination	All cells are tested negative for mycoplasma contamination using PCR Mycoplasma Test Kit from AppliChem GmbH
Commonly misidentified lines (See ICLAC register)	None of the cell lines used in this study is listed in the database of commonly misidentified cell lines maintained by ICLAC.
